# Advances in Metal Halide Perovskite Memristors: A Review from a Co‐Design Perspective

**DOI:** 10.1002/advs.202409291

**Published:** 2024-11-19

**Authors:** Bowen Jiang, Xiang Chen, Xiaoxin Pan, Li Tao, Yuangqiang Huang, Jiahao Tang, Xiaoqing Li, Peixiong Wang, Guokun Ma, Jun Zhang, Hao Wang

**Affiliations:** ^1^ Hubei Yangtze Memory Laboratories Wuhan 430205 China; ^2^ Institute of Microelectronics and Integrated Circuits, School of Microelectronics Hubei University Wuhan 430062 China

**Keywords:** biomimetic application, mechanism, memristor, metal halide perovskite

## Abstract

The memristor has recently demonstrated considerable potential in the field of large‐scale data information processing. Metal halide perovskites (MHPs) have emerged as the leading contenders for memristors due to their sensitive optoelectronic response, low power consumption, and ability to be prepared at low temperatures. This work presents a comprehensive enumeration and analysis of the predominant research advancements in mechanisms of resistance switch (RS) behaviors in MHPs‐based memristors, along with a summary of useful characterization techniques. The impact of diverse optimization techniques on the functionality of perovskite memristors is examined and synthesized. Additionally, the potential of MHPs memristors in data processing, physical encryption devices, artificial synapses, and brain‐like computing advancement of MHPs memristors is evaluated. This review can prove a valuable reference point for the future development of perovskite memristors applications. In conclusion, the current challenges and prospects of MHPs‐based memristors are discussed in order to provide insights into potential avenues for the development of next‐generation information storage technologies and biomimetic applications.

## Graphical abstract

This work presents a thorough review of the metal halide perovskite (MHP) memristors, covering materials to applications. The design principles and critical factors affecting device performance are summarized. The mechanism of MHP memristors is also concerned with deeper insight into fundamental principles. The challenges and opportunities in the application areas of MHP memristors are also analyzed. This review will provide valuable guidance for researchers working in this field.

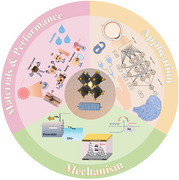

## 1 Introduction

Edge devices, cloud computing, and the Internet of Things (IoTs) are continuously advancing our lives towards greater intelligence.^[^ ^1^, ^2^, ^3^ ^]^ Nevertheless, enormous amounts of data necessitate more powerful computers for efficient processing. As chip feature sizes constantly approach their physical limit, it becomes increasingly difficult to enhance the processing power of computers, which prompts researchers to explore alternative devices and computer architectures.^[^ ^4^, ^5^, ^6^, ^7^ ^]^ The introduction of the memristor, a fourth passive device, has shown promise in overcoming the issue of data transfer in conventional computing systems.^[^ ^8^, ^9^, ^10^ ^]^ This issue, known as the “memory wall,” arises from the separation of computing and storage modules in the von Neumann system.^[^ ^11^, ^12^, ^13^ ^]^ Several reports have exhibited that memristors enable in‐memory computing with low power consumption, fast response, and higher integration,^[^ ^14^, ^15^ ^]^ which prompted researchers to investigate the application of memristors in the information field and achieve significant breakthroughs.

Due to the extensive data transfer between portable devices, data leakage has become a significant problem in the field of information security. Unlike software encryption, the use of encryption methods such as Physical Unclonable Functions (PUFs) contributes to the prevention of hacking.^[^ ^16^, ^17^ ^]^ In particular, since the random characteristics of the device cannot be easily simulated, the memristor‐based hardware security primitives could effectively increase the decryption time and provide information security.^[^ ^18^, ^19^, ^20^ ^]^ In addition to the random property that allows them to act as an encrypted source, extensive studies have demonstrated the abilities of memristors to exhibit nonlinearities and to respond to variations in the amplitude and frequency of input signals, which is similar to the capacity of neurons to tune synaptic transmission by potential differences generated by variations in the concentrations of neurotransmitters (K^+^ and Ga^+^, etc.).^[^ ^21^, ^22^, ^23^, ^24^, ^25^, ^26^, ^27^, ^28^ ^]^ This similarity allows the memristor to mimic the various biological forms of synaptic plasticity and, moreover, to mimic the memory‐learning behavior of the brain. Therefore, the memristor has emerged as a leading candidate for the realization of bionic devices and brain‐like computation. Additionally, memristors have garnered significant attention in the field of artificial neural networks (ANNs).^[^ ^29^, ^30^, ^31^, ^32^, ^33^, ^34^ ^]^ In recent years, there has been a significant amount of research conducted on memristor‐based neural networks, resulting in notable advancements in device materials, algorithm optimization, and recognition and classification.^[^ ^35^, ^36^, ^37^, ^38^, ^39^ ^]^ However, the majority of these achievements have been limited to software‐based simulations, and the development of a fully hardware‐implemented memristor‐based neural network remains a significant technological challenge. Fortunately, the team led by Huaqiang Wu at Tsinghua University has successfully developed the world's first memristor chip capable of on‐chip learning. This achievement, made possible by the fully hardware‐implemented memristor convolutional neural network (CNN) technology, confirms the viability of using memristors in neural networks.^[^ ^40^ ^]^ Furthermore, it encourages other researchers to investigate various device materials and explore wider applications in the field of memristor research.

Various materials, including oxide materials, sulfide materials, organic polymers, and perovskite, have exhibited the RS phenomenon.^[^ ^41^, ^42^, ^43^, ^44^, ^45^, ^46^ ^]^ Regrettably, the implementation of memristors has been hindered by constraints such as the inadequate environmental stability of organic materials, the intricate process of sulfur compounds, and the elevated preparation temperature of oxide materials.^[^ ^45^, ^47^ ^]^ Metal halide perovskites (MHPs) have recently attracted considerable interest due to their superior flexibility, structural adjustability, low power consumption, and excellent photoelectric response.^[^ ^48^, ^49^, ^50^, ^51^, ^52^, ^53^ ^]^ MHPs are characterized by the molecular formula ABX_3_, where the A represents an organic or inorganic monovalent cation such as MA^+^ (asmethylammonium), or FA^+^ (formamidine) located at the apex angle of the cubic lattice. Site is occupied by a divalent cation—either Pb^2+^ or Sn^2+^, positioned at the center of the cubic lattice. Meanwhile, the X represents the halogen ions (Cl^−^, Br^−^, or I^−^) that are located at the face‐centered positions. The substitution of organic methylamine with an inorganic ion, such as cesium or rubidium, results in the formation of a fully inorganic perovskite. Moreover, the perovskite structure permits the substitution of both the B and X sites, thereby illustrating the structural adaptability of MHP materials. Due to their numerous advantages, MHPs have emerged as highly promising candidates for a variety of optoelectronic applications, including solar cells, photodetectors, light‐emitting diodes (LEDs), and field‐effect transistors (FETs).^[^ ^54^, ^55^, ^56^, ^57^, ^58^, ^59^, ^60^, ^61^, ^62^ ^]^ Additionally, research has indicated that MHP materials are well‐suited for memristors.^[^ ^63^ ^]^ In particular, the combination of perovskite materials' photosensitivity with memristors allows for the creation of a novel class of perovskite‐based photoelectric memristors, with potential applications that extend beyond the capabilities of conventional oxide memristors.

MHPs memristors have attracted great attention as new application areas of perovskite materials after the first report of MAPbI_3‐x_Cl_x_‐based memristors by Yoo et al. in 2015.^[^ ^64^ ^]^ Later, in 2017, Kim et al.^[^ ^65^ ^]^ and Zhu et al.^[^ ^66^ ^]^ initially described the ionic vacancy migration motion inside MHPs, which provided an explanation of the resistive mechanism of perovskite memristors. Subsequently, additional research on perovskite memristors was conducted, including investigations into lead‐free perovskite,^[^ ^67^, ^68^ ^]^ inorganic perovskite,^[^ ^69^ ^]^ perovskite quantum dots,^[^ ^70^, ^71^ ^]^ perovskite nanocrystals^[^ ^72^, ^73^ ^]^ and other perovskite memristors with varying compositions, dimensions, and structures, as well as studies on the optimization of perovskite memristor performance through passivation^[^ ^74^, ^75^ ^]^ and doping.^[^ ^76^ ^]^ And in 2016, Xu et al.^[^ ^77^ ^]^ simulated synaptic plasticity with perovskite memristors which pioneered the exploration of artificial synaptic devices based on MHPs memristors. Numerous applications of MHPs memristors in neuromorphic computing have been reported subsequently. In 2020, Zhu et al. demonstrated a memristor‐based reservoir computation (RC) system that enables effective neural signal analysis with high spatiotemporal accuracy and potentially closed‐loop feedback control.^[^ ^78^ ^]^ In the same year, Gu et al.^[^ ^79^ ^]^ prepared hemispherical retinas based on high‐density perovskite nanowire arrays prepared by gas‐phase method. This work not only fabricated highly integrated optoelectronic devices on non‐planar substrates, but also inspired subsequent research on artificial bionic devices. Subsequently additional perovskite‐based synaptic devices and neural system computations have been successively reported. Furthermore, additional research has been conducted on perovskite‐based synaptic devices and neural system computations. For example, further studies on PVK‐based RC system by Yang et al.^[^ ^80^ ^]^ in 2022, and studies on two forms of perovskite memristors, drift and diffusion, reported by John et al.^[^ ^81^ ^]^ and Wang et al.^[^ ^82^ ^]^ in 2022 and 2023, respectively.

Given the nearly decade‐long history of perovskite memory resistor development, there is a substantial body of literature comprising review articles on perovskite memristors, offering diverse perspectives on this topic. Fang et al. provided a summary of the development of lead‐based to lead‐free perovskite memristors from the perspective of the toxicity and stability of perovskites,^[^ ^47^ ^]^ and Gogoi et al. provided an overview of the development and application perspectives of flexible perovskite‐based memristors.^[^ ^83^ ^]^ They summarized and analyzed from the perspective of device characterization. Additionally, there are also review articles that describe things from the perspective of material classification. For instance, Guan et al. and Liu et al. summarized the applications of low‐dimensional perovskite materials and nanostructured perovskite materials in the field of memristors, respectively.^[^ ^84^, ^85^ ^]^ Additionally, there are also reviews that focus on applications, such as overviews on synaptic devices,^[^ ^86^ ^]^ neural system computation,^[^ ^87^ ^]^ RC,^[^ ^88^ ^]^ artificial intelligence,^[^ ^89^ ^]^ etc. Unlike these published review articles, in our review, we start from the point of view of optimizing device performance, from the device working mechanism, material selection, device structure, and different memristor application fields are more comprehensive summary and discussion. Firstly, the conduction mechanisms based on MHPs memristors were discussed, including the CFs model, interface‐type RS, and charge trapping/detrapping. Despite the mechanism have been discussed in the reported reviews of perovskite memristors, we aim to provide a comprehensive overview of the mechanism of perovskite memristors, with an emphasis on the equipment utilized in these reports for demonstrating the mechanism, which has not yet been overviewed in the previous reviews. Moreover, we then provide an overview of the optimization strategies of researchers on perovskite memristors in recent years. Similarly, numerous reviews of perovskite memristors with excellent performance, such as the previously mentioned reviews from a low‐dimensional material perspective or from the perspective of device parameters,^[^ ^90^ ^]^ but a comprehensive overview encompassing diverse optimization strategies focus on enhancing the performance of MHPs‐based memristors remains absent. We analyze and demonstrate how optimization strategies such as interface passivation, crystal modulation, dimensional modulation, and doping influence various aspects of the performance of MHPs memristor including storage window, endurance, retention time, switching speed, power consumption, etc. In part four, we present and discuss in detail the potential applications of MHPs memristors such as information computing, information security, bionic synapses, and ANNs from multiple perspectives. In contrast to traditional Complementary‐Metal‐Oxide‐Semiconductor (CMOS) logic gates, a solitary memristor is capable of performing logic operations, such as “and” and “or”. Furthermore, the random character of the resistance fluctuation in the MHPs memristor may also be exploited in the domain of information security. We provide a comprehensive analysis of MHPs memory resistors simulating different synaptic behaviors, focusing on their potential application in bionic synapses. Finally, we discuss the utilization of MHPs synaptic devices in brain‐like computational neural networks and bionic visual neural networks. Finally, we present an overview of the potential obstacles and prospects for the future advancement of MHP‐based memristors.

## 2 RS Mechanism of MHPs Memristor

As research on perovskite memristors continues to evolve, the RS mechanism of these devices has begun to emerge as a prominent area of investigation. A comprehensive understanding of the RS mechanism based on MHPs memristors is crucial for the selection of perovskite materials, the optimization of films, and the improvement of performance. The existence of a significant hysteresis in the current‐voltage (*I‐V*) curve of MHPs devices caused by charge trapping or ionic transport, allows MHPs to be well‐suited for application in memristors.^[^ ^91^, ^92^ ^]^ Several RS mechanisms have been proposed to explain the hysteresis in halide perovskites, including the conductive filaments (CFs) model,^[^ ^26^, ^70^, ^93^, ^94^, ^95^, ^96^ ^]^ interface‐type RS,^[^ ^97^, ^98^, ^99^, ^100^ ^]^ and charge trapping/detrapping.^[^ ^101^, ^102^ ^]^ Various characterization techniques have been used to support the RS mechanism of perovskite memristors. These methods include scanning electron microscopy (SEM),^[^ ^103^, ^104^ ^]^ energy dispersive X‐ray spectroscopy (EDS),^[^ ^103^ ^]^ transmission electron microscopy (TEM),^[^ ^105^ ^]^ X‐ray photoelectron spectroscopy (XPS),^[^ ^106^ ^]^ Kelvin probe force microscopy (KPFM),^[^ ^72^ ^]^ atomic force microscope (AFM),^[^ ^72^, ^107^ ^]^ and impedance spectrum analysis.^[^ ^102^, ^108^ ^]^ In this section, we will specifically focus on the RS mechanism of MHPs and summarize the widely recognized RS mechanisms currently employed to elucidate halide perovskite memristors.

### 2.1 CFs Model

**Figure 1:**
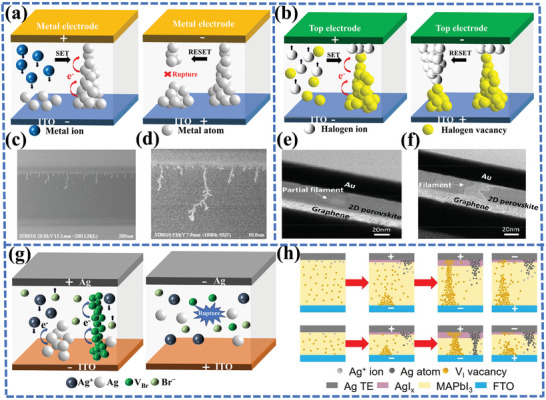
a) RS mechanism of memristor under Active metal CFs mechanism. b) RS mechanism of memristor under Halide vacancy filaments induced by ion migration mechanism. c) Top view SEM image of the Ag/PMMA@CsPbI_3_/Ag device. d) Magnified SEM image of the region near the grounded electrode.^[^ ^104^ ^]^ Copyright 2020, American Chemical Society. e) TEM image of an area showing a partial filament. f) TEM image showing the filament shape (white dotted line) with a larger diameter (≈30 nm) close to graphene side and a smaller diameter (≈15 nm) close to Au side.^[^ ^105^ ^]^ Copyright 2017, American Chemical Society. g) RS mechanism of memristor under coexistence between active metal CFs and halide vacancy filaments. h) Double‐filament model of resistive switching behaviors in the fluorine‐doped tin oxide (FTO)/MAPbI_3_/Ag memory device.^[^ ^120^ ^]^ Copyright 2018, American Chemical Society.

In recent years, the CFs model has been widely reported in perovskite memristors.^[^ ^109^, ^110^, ^111^ ^]^ According to this model, the formation/rupture of CFs facilitates the device switching between a low resistance state (LRS) and a high resistance state (HRS). The CFs model is depicted in **Figure** **1**a,b. The application of an external voltage results in the formation of CFs within the active layer (perovskites), which function as channels for large currents, thereby enabling the device to operate in the LRS. Conversely, if the external voltage changes in the opposite direction, the internal CFs rupture, and the memristor switches from the LRS to HRS. Numerous studies have demonstrated the existence of a multitude of CFs models for MHPs memristors with different device structures. Generally, MHPs memristor CFs models are categorized into two types: active metal CFs and halide vacancy CFs induced by ion migration. Interestingly, there is also a distinct scenario where active metal CFs and halide vacancy filaments compete with one another.

#### 2.1.1 CFs with Active Metal

Memristors based on the active metal filament model commonly utilize active metals (such as Ag and Cu) for the top electrode and inert metals (such as indium tin oxide (ITO) and Pt) for the bottom electrode. The formation/fracture of CFs primarily occurs due to the redox reactions of metals with electrochemical activity and the migration of the corresponding cations.^[^ ^112^ ^]^ For instance, in the case of Ag filament formation/fracture, the application of a positive bias voltage to the top electrode causes the oxidation of Ag to Ag^+^, which then migrates toward the bottom electrode under the influence of an electric field. This migration ultimately results in the reduction of Ag^+^ to Ag atoms at the bottom electrode. With the continuous redox reaction, an increasing amount of Ag is involved in the reaction and accumulates, which eventually forms Ag CFs connecting the two electrodes, prompting the device to change from the HRS to LRS. On the other hand, the Ag CFs are dissolved and transformed into Ag^+^ when a reverse bias is applied, causing the device to revert to the HRS.

Regarding active metal CFs, it is worth noting that the LRS is usually unaffected by the size of the device,^[^ ^65^, ^113^ ^]^ and the resistance of the LRS shows positive linear characteristics with temperature.^[^ ^112^, ^113^ ^]^ Additionally, the formation/fracture diagram of metal CFs is depicted in Figure 1a. The presented data provide indirect evidence for the existence of metal CFs. Xu et al. fabricated a two‐terminal device based on PMMA@CsPbI_3_ membrane using Ag electrodes to directly observe the Ag CFs.^[^ ^104^ ^]^ They subjected the device to a voltage bias with a 1 mA compliance current for 12 h. They discovered that multiple dendritic microstructures of Ag metal grew and accumulated near the grounded electrode region (Figure 1c,d) through SEM. Therefore, this basic device effectively demonstrated the whole oxidation process of the active anode Ag and the subsequent migration of Ag^+^ inside the PMMA@CsPbI_3_ membrane. Additionally, when the Ag electrode was replaced by the Au electrode, there was no observation of any microstructure through the same bias process. Similarly, the existence of Ag CFs was also validated by Wang et al. using SEM measurements and EDS analysis.^[^ ^103^ ^]^ They compared parietal SEM images of the transverse structural RRAM after two types of stimulation (low‐voltage bias stimulation and SET operation). Under the action of applied voltage bias, numerous Ag filaments were formed between the anode and cathode. Furthermore, the distribution of Ag and Pb was measured along the silver filament and in randomly chosen regions. The EDS results indicated a significant concentration of Ag in the vicinity of the filament, while a considerably lesser quantity of Ag was detected in the randomly chosen region. A recent study published by Luo et al. provides a verification process for systematic characterization of metallic CFs.^[^ ^114^ ^]^ They utilized in situ PL imaging microscope technology to observe the formation and rupture of CFs. This provides a visual characterization method for the study of the resistance transition process of perovskite memristors. In addition, researchers have also proposed that active metals like Cu CFs.^[^ ^115^ ^]^

#### 2.1.2 Halide Vacancy Filaments Induced by Ion Migration

In contrast to active metal electrodes, inert metal electrodes (e.g., Au) tend not to participate in redox reactions. Nevertheless, similar RS phenomena have been observed in devices with inert metal electrodes.^[^ ^116^ ^]^ These phenomena primarily arise from ionic motion due to the various types of point defects in perovskite, including vacancies (V_Pb_, V_MA_, V_I_), interstitials (Pb_i_, MA_i_, I_i_), and antisites (Pb_I_, I_Pb_) In this context, A_B_ indicates that substitution of A by B.^[^ ^117^ ^]^ Among these mobile ions, the activation energy of halogen vacancy is relatively low and capable of easily moving along the soft inorganic octahedral lattice edges of the perovskite structure.^[^ ^118^, ^119^ ^]^ Therefore, the migration and redistribution of halogen ions and halogen vacancies under an external electric field result in the formation and fracture of filaments. As the applied voltage rose, halogen vacancies gradually accumulated at the cathode electrode, leading to the formation of halogen vacancy filaments within the perovskite layer.

The mechanical exfoliation was employed by Tian et al. to obtain high‐quality two‐dimensional (2D) (PEA)_2_PbBr_4_ films, constituting the Au/2D perovskite/graphene sandwich structure. They observed nano‐CFs with a diameter of around 20 nm in 2D perovskite using cross‐sectional TEM (Figure 1e,f) and they found evidence suggesting the production of Br vacancies due to the migration of Br ions.^[^ ^105^ ^]^ These experimental findings provide significant support for the halide vacancy filaments concept. Following the characterization of metal‐conducting filaments via in‐situ PL, Luo et al. also characterized halogen vacancy conducting filaments with Au electrode devices.^[^ ^114^ ^]^ The photoluminescence of perovskite exhibits a time‐dependent change in response to voltage bursts, undergoing a transition from positive to negative with time. This phenomenon is indicative of the formation of halogen vacancy filaments, as evidenced by the concomitant decrease in current.

#### 2.1.3 Competition Between Active Metal CFs and Halide Vacancy Filaments

Interestingly, although the validity of the above two types of filament models has been proven, the actual formation of CFs is probably the result of competition/coexistence between active metal CFs and halide vacancy filaments (Figure 1g). In order to investigate the underlying mechanisms of two types of filaments, Sun et al. fabricated devices based on the structure of Pt/CH_3_NH_3_PbI_3_/Ag with different thicknesses (about 300 nm, 240 nm, 150 nm, and 90 nm). They revealed the RS behavior of the devices resulted from the competition between Ag and V_I_ filaments as shown in Figure 1h.^[^ ^120^ ^]^ RS devices relying on Ag^+^ migration typically require an electric field of at least 10^7^ V m^−1^,^[^ ^121^ ^]^ which presents a significant challenge for the formation Ag CFs in perovskite devices that are 300 nm thick. Consequently, V_I_ CFs become the dominant factor. However, when the thickness of the MAPbI_3_ film is reduced to approximately 90 nm, both types of CFs can coexist.

### 2.2 Interface‐Type RS

Instead of the redox reaction of metal‐conducting filaments, the ion‐electron coupling could introduce interfacial doping around the electrodes as an additional mechanism of resistive switching.^[^ ^122^ ^]^ Manipulation of the external electric field could induce a transition in the resistive state of the device through modification of the charge injection and modulation barriers of ion accumulation and dipole formation at the perovskite/transport layer interface caused by the physical and chemical reactions occurring at the interface.^[^ ^123^ ^]^ Specifically, the drift and diffusive motion of ions within perovskite material, driven by both internal and external electric fields, could induce self‐p or n doping at the interface between perovskite and the transport layer. This would modulate the carrier injection barrier, thereby influencing the conductivity.^[^ ^124^, ^125^ ^]^

**Figure 2:**
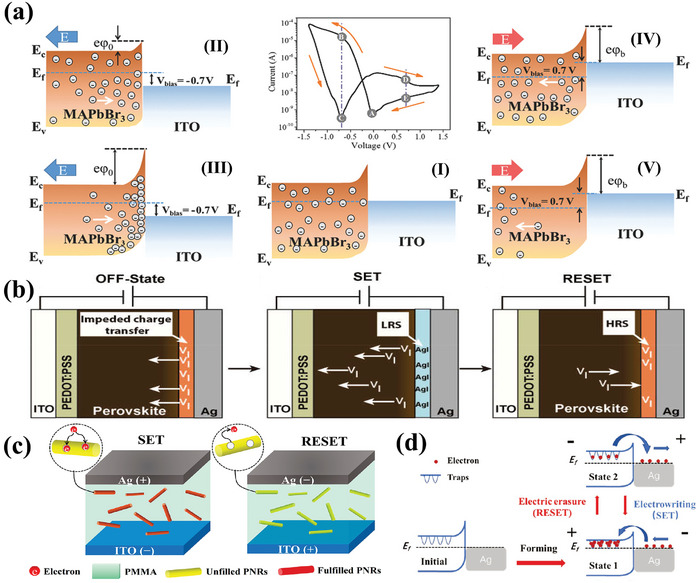
a) Schematics of the dynamic ion migration near the perovskite/ITO interface during the RS switching. (I), (II), (III), (IV), and (V) are several specific points in the *I*–*V* curve, which correlate with the modified ion distribution and band alignment at the perovskite/ITO Schottky junction during the bias scan.^[^ ^97^ ^]^ Copyright 2018, John Wiley and Sons. b) Mechanism explanation diagrams for the three states.^[^ ^108^ ^]^ Copyright 2020, American Chemical Society. c) The SET and RESET progress occurred via trapping and detrapping of the electrons by PNRs in the ITO/PNRs@PMMA/Ag structure.^[^ ^127^ ^]^ Copyright 2021, American Chemical Society. d) Schematic of RS behavior mechanisms for the ITO/Cs_3_Bi_2_Br_9_/Ag devices.^[^ ^128^ ^]^ Copyright 2022, John Wiley and Sons.

It has been demonstrated that charged vacancy defects in perovskites accumulate at the interface due to external electric fields, thus altering the potential barrier at the interface. As shown in **Figure** **2**a, point (I) indicates the existence of a multitude defects within the perovskite layer situated near the perovskite /ITO interface, even in the absence of any external bias. When a negative bias is applied to the Au electrode, the junction current experiences a quick initial rise due to the positive bias of the rectifying diode as shown in point (II) of Figure 2a. Subsequently, with the negative bias increased, the MA^+^ ions drift toward the positive electrode, and correspondingly MA vacancies move toward the ITO electrode and accumulate along the perovskite/ITO interface (point (III)). The accumulation of charges leads to an increase in the Schottky barrier height, which in turn causes a sharp drop in device current.^[^ ^97^ ^]^ When a reverse bias is applied (at points (IV)/(V)), the diode is reverse bias conditions, causing a relatively low current.

Similarly, Han et al. proposed that the accumulation of Sn vacancies at the Au/CsSnI_3_ interface led to a reduction in the width of the p‐type perovskite layer depletion region, causing a decrease in both the Schottky barrier and contact resistance.^[^ ^69^ ^]^ As the depletion layer width decreased, electrons were able to tunnel through the thin Schottky barriers more easily, resulting in a decrease in contact resistance, and the device transforms from HRS to LRS in the meantime. Conversely, the decrease of Sn vacancies at the interface causes a rise in the width of the depletion layer, resulting in higher Schottky barriers and increased contact resistance, and the device transforms to HRS. Solanki et al. presented the physical/chemical reaction mechanism at the interface of perovskite/Ag by utilizing scanning Kelvin probe microscopy (SKPM), conductive atomic force microscopy (c‐AFM), and impedance spectroscopy.^[^ ^108^ ^]^ As shown in Figure 2b, the device exhibited three distinct resistive states. The presence of iodine vacancy (V_I_) at the interface inhibited the charge transfer from Ag to the perovskite layer, resulting in the device entering the OFF state. However, when a positive voltage was applied, the I^−^ ions reacted with oxidized Ag^+^ to form a thin layer of AgI, which facilitated efficient charge transfer, resulting in the formation of LRS through the SET process. At the opposite polarity of the applied bias, V_I_ migrated in the opposite direction and eventually accumulated at the interface of perovskite/Ag, leading to HRS corresponding to the RESET process. In addition, these reaction processes were reversible.

### 2.3 The Charge Trapping and Detrapping

The perovskite materials may exhibit a high concentration of defects on the surface or within their structure, which can act as traps for capturing electrons or holes.^[^ ^126^ ^]^ Under forward bias, these defects are filled with electrons or holes, resulting in the formation of favorable p‐type and n‐type contacts at the interface. This transition from HRS to LRS facilitates the operation of the devices. In contrast, the trap is gradually emptied, resulting in the device returning to HRS.

Das et al.^[^ ^127^ ^]^ constructed the ITO/perovskite nano‐rods PNRs@PMMA/Ag structure and they suggested that the switching mechanism could be explained in association with charge trapping/detrapping, which is assisted by the defects/traps present inside the mixed halide PNRs (Figure 2c). When a positive voltage was applied, the electrons were able to tunnel through the ITO/PMMA barrier due to the reduced barrier at the ITO/PMMA interface and were trapped in the PNRs. As all the traps were gradually filled, the injected electrons could easily pass through, forming a conductive path and the device was transferred from HRS to LRS. Conversely, the electrons that had been captured were extracted from the capture center and transferred to the ITO. This resulted in a complete disconnection of the charge transfer channel due to the absence of occupied capture sites (RESET process). Similarly, the interface effect based on the electron trapping/detrapping processes of ITO/Cs_3_Bi_2_Br_9_/Ag structure was also proposed for the RS behavior.^[^ ^128^ ^]^ As shown in Figure 2d, in the Ag/Cs_3_Bi_2_Br_9_ interface, the bromine in Cs_3_Bi_2_Br_9_ film is captured by the Ag electrode to form Br vacancies, which are known as electron‐traps (initial state). During SET process, the electrons progressively occupy the electron traps located at the interface. Meanwhile, the Fermi energy level of Cs_3_Bi_2_Br_9_ shifted in the direction of the valence band, which caused the reduction of the Schottky barrier (state 1). The application of a reverse electric field resulted in the release of electrons from their traps and their migration towards the Ag electrode, leading to the reestablishment of the energy barrier (State 2).

A comprehensive list of studies examining the RS mechanisms of perovskite memristors is provided in **Table** **1**. It is clear from the numerous studies that have been conducted that the RS behaviors of perovskite memristors are closely related to the movements of ions within the device. To demonstrate the trend of these internal ionic motions, various direct and indirect methods have been proposed. For example, EDS scanning, in‐situ PL, TEM, and other characterization methods, as well as the dependence of device performance on area, temperature, etc. Nevertheless, the intricate nature of the ion‐electron dynamics within the perovskite structure has resulted in a paucity of comprehensive theoretical understanding. Consequently, further in‐depth investigation is imperative to elucidate the internal ion dynamics of the perovskite memristor. In addition, there are also groups studying the synaptic dynamics of MHPs from the perspective of impedance transient dynamics, such as Bisquert's group, which has successively analyzed the frequency and time domains of the perovskite memristor,^[^ ^129^ ^]^ as well as capacitance and inductance effects.^[^ ^130^ ^]^ These studies contribute to the theoretical understanding of the MHPs memristor mechanism, the realization of artificial synapses and the construction of neural networks. However, there are relatively few reports on the transient dynamics of MHPs memristors, and numerous follow‐up studies are needed.

**Table 1:** RS mechanisms based on MHPs memristor.

Device structure	Mechanism	Refs.
Si/SiO_2_/Ti/Pt/(BzA)_2_CuBr_4_/PMMA/Ag	Formation/Rupture of Ag CFs	[98]
Si/SiO_2_/Ti/Ag/CsPbBr_3_‐single crystal film/Ag	Formation/Rupture of Ag CFs	[99]
FTO/PMMA@CsPbI_3_/Ag	Formation/Rupture of Ag CFs	[104]
FTO/ MAPbI_3−_ *_x_* Cl*_x_*/Ag	Formation/Rupture of Ag CFs	[113]
Si/SiO_2_/Ti/Pt/CsSnI_3_/ultra‐thin PMMA/Ag	Formation/Rupture of Ag CFs	[69]
Si/SiO_2_/Ti/Pt/CsSnI_3_/PMMA/Ag	Formation/Rupture of Ag CFs	[67]
ITO/MA_3_Bi_2_I_9_/Cu	Formation/Rupture of Cu CFs	[115]
ITO/CsPbBr_3_ QDs/PMMA/Ag	Formation/Rupture of Ag+V_Br_ CFs	[71]
PET/ITO/PMMA/CsPbBr_3_ QDs/PMMA/Ag	Formation/Rupture of Ag+V_Br_ CFs	[103]
PET/ITO/PMMA/CsPbBr_3_ QDs/PMMA/Au	Formation/Rupture of V_Br_ CFs	[103]
Si/SiO_2_/Au/MAPbI_3_/Au	Formation/Rupture of V_I_ CFs	[95]
ITO/MAPbBr_3_ (350 nm)/Au	Formation/Rupture of V_Br_ CFs	[97]
ITO/MAPbI_3_ (500 nm)/Au	Formation/Rupture of V_I_ CFs	[97]
ITO/SnO_2_/α‐FAPbI_3_/[CNBmim]Cl/Au	Formation/Rupture of V_I_ CFs	[100]
Au/(PEA)_2_PbBr_4_ SC/graphene	Formation/Rupture of V_Br_ CFs	[105]
ITO/MAPbI_3_/Au	Formation/Rupture of V_I_ CFs	[119]
ITO/MAPbI_3_:PVAm·HI/Al	Formation/Rupture of V_I_ CFs	[131]
ITO/MAPbI_3_‐supramolecular framework/Al	Formation/Rupture of V_I_ CFs	[132]
Si/Pt/CsPbBr_3_/Ag	Formation/Rupture of Ag+V_Br_ CFs	[133]
Au/(PEA)_2_PbI_4_ single crystals/Au	Formation/Rupture of V_I_ CFs	[134]
ITO/α‐CsPbI_3_/Au	Formation/Rupture of V_I_ CFs	[135]
Pt/MAPbI_3_/Ag	Competition between Ag and V_I_ CFs	[120]
ITO/MAPbBr_3_ (1000 nm)/Au	Interface type (V_MA_ modifies schottky barrier)	[97]
ITO∖PEDOT:PSS∖(PEA)_2_(MA)_n‐1_Pb_n_I_3n+1_∖PCBM∖Ag (n = 1,3,5,7,∞)	Interface type (interface reaction)	[108]
Ag/ MAPbI_3_ micro‐/nanofibers/Ag	Interface type (interface reaction)	[136]
Si/SiO_2_/Ti/Pt/CsSnI_3_/Au	Interface type (V_Sn_ changes the depletion width)	[69]
FTO/MAPbI_3−_ *_x_* Cl*_x_*/Au	Charge trapping/detrapping (injection/ejection of holes at the interfacial hole trapping centers)	[101]
FTO/MAPbI_3_/Au	Charge trapping/detrapping (trapping/detrapping of the electrons in bulk defects and interface)	[102]
ITO/RbPbI_2.4_Cl_0.6_@PMMA/Ag	Charge trapping/detrapping (trapping/detrapping of the electrons by RbPbI_2.4_Cl_0.6_ nanorods)	[127]
ITO/ Cs_3_Bi_2_Br_9_/Ag	Charge trapping/detrapping (trapping/detrapping of the electrons by V_Br_)	[128]
Au/(PEA)_2_SnI_4_/Au transistors (Si/SiO_2_ Gate)	Charge trapping/detrapping (Sn vacancies captured the photogenerated electrons)	[137]
ITO/ Cs_2_AgBiBr_6_@PMMA/Au	Charge trapping/detrapping (trapping/detrapping of the electrons by trap centers)	[138]

## 3 Performance Optimization Strategies of MHPs Memristor

MHPs memristors have attracted significant interest from researchers due to their unique microstructures and integration abilities, which are distinguished by excellent ON/OFF ratios, outstanding stability, and low cost. As early as 2015, the first MHPs memristor with typical bipolar RS behavior prepared by Yoo et al. was introduced with the structure of FTO/MAPbI_3‐x_Cl_x_/Au.^[^ ^64^ ^]^ The curves of the devices remained almost consistent after the first and the hundredth operation, indicating the excellent repeatability and stability of the devices. In addition, all devices exhibited low operating voltages (*V*_SET_ = 0.8 V, *V*_RESET_ = −0.6 V), stable endurance (>100 cycles) and long retention time (>10^4^ s) were available. Subsequently, the Au top electrode was with Ag, resulting in the emergence of novel properties, including mimetic switching characteristics and synaptic behavior, which further triggered the interest of the researchers in applying MHPs to neuromorphic computing devices.^[^ ^113^ ^]^ Despite the lack of outstanding performance, Yoo et al. initiated the development of MHPs memristor research. Nowadays, various types of MHPs memristors are applied in the development of information storage technologies for computers.

In this section, we present the current progress in optimizing the performance of MHPs memristors through various techniques such as: passivation, adapting the crystal structure or dimension, doping, etc. In addition, the performance of the most representative devices in the development of MHPs memristors in recent years is comprehensively summarized (**Table** **2**).

**Table 2:** Comparison of MHPs memory performance.

Device Structures	ON/OFF ratio	*V*_SET_/*V*_RSET_ [V]	*I*_cc_ [A]	Endurance	Retention [s]	Stability	Refs.
FTO/MAPbI_3−x_Cl_x_/Au	10	+0.8/−0.6	10^−2^	10^2^	10^4^	2 weeks Air, RT	[64]
FTO/MAPbI_3−x_Cl_x_/Ag	10^2^	+1.5/−1	10^−3^	10^3^	10^4^	2 months RT, ≈30％ Humidity	[113]
FTO/TiO_2_/MAPbCl_x_I_3‐x_/M‐Al	1.9 × 10^9^	+0.25/−2	10^−6^	/	/	/	[112]
Si/SiO_2_/Ti/Pt/(PEA)_2_Cs_3_Pb_4_I_13_/Ag	10^9^	+0.2/−0.1	10^−3^	2 × 10^2^	2 × 10^3^	2 weeks ambient condition	[139]
ITO/RbPbI_3−x_Cl_x_/Ag	10^2^	+2/−1.5	10^−7^	623	/	/	[140]
ITO/MAPbI_3−x_Cl_x_/BAPbI_3_/Al	10^3^	+0.79/−0.6	10^−3^	3 × 10^2^	10^4^	/	[141]
Al@MAPbI_3_/Al	10^6^	+1.66/−0.47	10^−1^	5 × 10^2^	10^4^	/	[142]
Si/SiO_2_/Ti/Pt/CsPbI_3_/ PMMA/Ag	10^6^	+0.2/−0.1	10^−3^	3 × 10^2^	/	/	[67]
ITO/Y‐MAPbI_3_/Al	10^3^	+0.5/−0.5	10^−2^	3 × 10^3^	10^4^	/	[143]
Si^++^/SiO_2_Blend‐II/(PEA)_2_PbBr_4_/C_8_‐BTBT/Au^a)^	10^4^			10^4^	5.5 × 10^4^	/	[144]
ITO/MAPbI_3_/ZnO/Au	10^2^	+1/−0.6	10^−3^	/	/	30 days Air at RT, 50–60% Humidity	[145]
ITO/MAPbI_3_/Au	10	+4/−3	10^−2^	10^3^	10^5^		[146]
Si/Pt/BA_2_MA_n_Pb_n_I_3n+1_/Ag	10^7^	+0.4/−0.6	10^−2^	250	/	Operating at 80 °C	[147]
ITO/MAPbI_3−x_Cl_x_/BAI/Al	10^6^	+0.15/−0.5	10^−5^	60	7 × 10^3^	/	[148]
Si/Pt,Ti/MAPbI_3_/PEA_2_PbI_4_/PMMA/Ag	10^6^	+0.18/−0.14	10^−3^	2.7 × 10^3^	/	28 days RT	[149]
Ag/MAPbCl_3_ NWs/Al	10^7^	+3/−2.5	10^−1^	3 × 10^6^	/	/	[150]
ITO/PET/3D‐SF/Al^b)^	10^5^	+2.3/−0.42	10^2^	5 × 10^2^	10^4^	/	[132]
FTO/MAPbI_3_/Al	10	+1/−1	10^−3^	5 × 10^2^	/	/	[151]
FTO/PVA/STPT/Ag^c)^	10^3^	+2.3/−0.5	10^−1^	/	10^5^	/	[152]
ITO/Cs_3_Bi_2_Br_9_/Ag	10	+1/−0.5	10^−3^	3.2 × 10^3^	10^3^	/	[128]
ITO/Cs_2_NaBiI_6_/Al	10^2^	−0.2/2	10^−1^	2 × 10^2^	10^4^	90 days Air at 130 °C	[153]
ITO/SnO_2_/Cs_2_AgBiBr_6_/NiOx/Ag	50	+0.7/−0.7	3 × 10^−2^	3 × 10^2^	10^3^	/	[154]
ITO/Cs_3_Sb_2_I_9_/Al	10^4^	+0.4/−3.2	10^−2^	10^2^	10^4^	/	[155]
ITO/MAPbI_3_/Al	10^3^	+3/−3	10^−4^	3 × 10^2^	/	60 days 45%‐55% Humidity	[156]
ITO/MAPbI_3_:ADNH_3_I/Al^d)^	10^8^	−0.3/+2	10^−1^	2 × 10^3^	10^4^	30 days 23–28 °C 60% Humidity	[157]
ITO/MA_3_Sb_2_Br_9_/PMMA/Ag	10^2^	−0.2/+0.45	10^−2^	3 × 10^2^	10^4^	/	[158]
ITO/MAPbI_3_/MoO_3_/Ag	10^2^	+0.9/−1.2	10^−1^	10^3^	1.1 × 10^3^	/	[114]
ITO/MAPbI_3_/MoO_3_/Ag	10^2^	+0.9/−1.2	10^−1^	10^3^	1.1 × 10^3^	/	[114]
ITO/CsPbBr_3_/Au	10	+0.29/−0.22	10^−1^	4 × 10^2^	4 × 10^2^	/	[159]
ITO /MAPbI_3–x_Cl_x_/PEASCN /Al	10^3^	+0.6/−0.7	10^−3^	10^3^	>10^4^	150 days Air at RT>20%	[160]
ITO/CsPb(Br_1−x_I_x_)_3_ QDs/Au	10^3^	+0.92/−3.01	/	10^2^	/	/	[161]
Au/Cs_2_AgBiBr_6_@PMMA/ITO	10^4^	+1.5/−1.5	10^−4^	10^3^	10^4^	/	[138]
Pt/FAPbI_3_/Ag	10^6^	+0.17/−0.19	10^−3^	2000	/	30 days	[162]
Au/MAPbI_3_:Ag/Au	10^5^	−1.2/+1	10^−3^	/	10^3^	/	[76]
ITO/α‐CsPbI_3_/Au	10^4^	+0.8/−0.6	10^−4^	1000	10^4^	15 days	[135]
ITO/PEDOT:PSS/MAPbI_3_/EgaIn^e)^	≈10^3^	+0.49/−3	10^−2^	10^4^	10^5^	9 days	[163]
ITO/PEDOT:PSS/MAPbI_3_/EgaIn^e)^	≈10^3^	+0.49/−3	10^−2^	10^4^	10^5^	9 days	[163]
ITO/PEDOT:PSS/PEACl‐MASnI_3_/In‐Sn	8.5 × 10^3^	+0.5/−4	10^−2^	2000	2 × 10^4^	4 days 373 K 80% RH Light 2h	[110]
Ag/CsPbBr_3_/Ag	10^9^	+0.8/−0.4	10^−3^	150	>10^4^	/	[99]
Graphite/ITO/PTAA/MAPbBr_3_/Graphite^f)^	10	+0.6/−2.3	/	10^3^	10^4^	/	[164]
Graphene/(PEA)_2_PbBr_4_/Au	10	+2.8/−1	10^−11^	100	10^3^	/	[105]
ITO/SnO2/α‐FAPbI_3_/ [CNBmim]Cl/Au^g)^	≈10^3^	+0.9/−2.5	10^−2^	550	10^4^	/	[100]
ITO/Ag/MAPbI_3_ QW/Au	≈107	+0.75/−0.5	/	10^6^	4.2 × 10^7^	18 months	[75]
ITO/PEDOT:PSS/PrPyr[PbI_3_]/PMMA/Ag^h)^	10^5^	+0.5/−3	10^−3^	450	10^4^	/	[165]
ITO/PEDOT:PSS/PrPyr[PbI_3_]/PMMA/Ag	10^6^	+0.7/−1	10^−3^	2000	10^5^	/	[166]
ITO/PEDOT:PSS/BnzPyr[PbI_3_]/PMMA/Ag^i)^	10^3^	+0.7/−3	10^−3^			/	[166]
ITO/PMMA/CsPbBr_3_ QD/PMMA/Ag	10^5^	+2.6/−2.8	/	5000	10^5^	365 nm, 0.153 mW cm^−2^ light	[103]
ITO/CsPbBr_3_/Ag	10^2^	+0.9/−1	10^−3^	10^4^	10^5^	/	[71]
FTO/MAPbI_3_/OA/W^j)^	10^2^	+2.5/−0.55	10^−2^	100	/	/	[74]

^a)^ 2,7‐Dioctyl[1]benzothieno[3,2b]benzothiophene(C_8_‐BTBT);

^b)^ 3D supramolecular framework (3D‐SF);

^c)^ Surface‐plasma‐treated inorganic halide perovskite (SPTP)/polyvinyl alcohol (PVA);

^d)^ 1‐Adamantylammonium (ADNH_3_^+^);

^e)^ Eutectic gallium and indium (EGaIn);

^f)^ Poly(triaryl amine) (PTAA);

^g)^ 1‐Cyanobutyl‐3‐methylimidazolium chloride ([CNBmim]Cl);

^h)^ Propyl pyridinium lead iodide (PrPyr[PbI_3_]);

^i)^ Benzylpyridinium lead iodide (Bnz)PbI_3_;

^j)^ Oleic acid (OA).

### 3.1 Passivation Optimization Strategy

Polycrystalline perovskite films prepared with conventional solution processes are vulnerable to massive defects at grain boundaries (GBs) and on the film surface. These defects could threaten the stability of perovskite devices.^[^ ^167^ ^]^ Numerous studies have demonstrated that interface passivation engineering with compositions and additives could effectively decrease the number of GBs and improve the quality of crystals and films.^[^ ^57^, ^168^, ^169^ ^]^ Generally, additives and passivators with ionic and coordination bonds such as guanidinium, amines, pyridine, thiophene, cyano, and sulfonate are considered to have interactions with uncoordinated sites on the surfaces of perovskite GBs and thin films to reduce defects.^[^ ^170^, ^171^ ^]^ Passivation engineering has likewise been shown to be a useful method of optimizing devices in the research of perovskite memristor devices.

**Figure 3:**
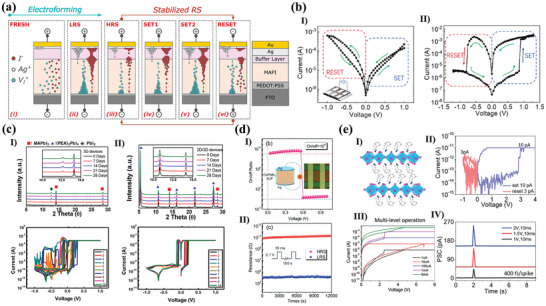
a) Schematic diagram of device and the electro forming and two‐step SET process mechanism under the applied voltage of the memristor with buffer layers.^[^ ^173^ ^]^ Copyright 2023, American Chemical Society. b) *I−V* curves of memory devices based on I) MAPbI_3−x_Cl_x_ and II) 2D/3D heterostructure perovskite.^[^ ^141^ ^]^ Copyright 2020, American Chemical Society. c) XRD spectra of the devices over time and *I‐V* curves of the devices after 28 days placement: I) 3D perovskite and (II) 2D/3D perovskite.^[^ ^149^ ^]^ Copyright 2020, American Chemical Society. d, I)The on/off ratio is plotted as a function of voltage. II) The retention of device by adding a series resistance of 10 kΩ.^[^ ^99^ ^]^ Copyright 2021, John Wiley and Sons. e, I) Illustration of the 2D layered structure of (PEA)_2_PbBr_4_; II) typical resistive switching curves for set and reset. The compliance current is 10 pA, and a 10^2^ on/off ratio is obtained; III) the multi‐level operations by setting the compliance current at different levels; IV) current response to 10 ms pulses with different voltage amplitudes. The power consumption is only 400 fJ per spike for the 1 V pulse.^[^ ^105^ ^]^ Copyright 2020, American Chemical Society.

Yang et al. indicated that devices passivated with P3HT exhibit hysteresis switching curves and a wider range of conductance modulation compared to non‐passivated devices, showing good stability, and reliability.^[^ ^172^ ^]^ They indicated that utilization of P3HT to passivate the positively charged defects on the perovskite surface reduces the number of migratable halogen ion vacancies, leading to slower processes of CFs formation and breakage, which has the potential to emulate the functionality of synapses. More generally, numerous research groups prefer to deposit a poly(methyl methacrylate) (PMMA) layer on the perovskite surface.^[^ ^69^, ^71^, ^104^ ^]^ The PMMA layer not only prevents penetration of the top active electrode into the perovskite and limits leakage currents, but also prevents the perovskite membrane from being exposed to moisture and oxygen in external environment. Additionally, Guerrero et al. also indicated that PMMA can not only provide a buffer layer acting as a barrier, but also as a storage pool for ions, when the external electric field is removed, as shown in **Figure** **3**a.^[^ ^173^ ^]^ In addition, Tao et al.^[^ ^100^ ^]^ and Cai et al.^[^ ^74^ ^]^ passivated perovskite surfaces with ionic liquids and oleic acid, respectively. The results of both studies indicated that the devices passivated in this manner exhibited a larger resistance ratio and more stable I‐V performance. It is noteworthy that both studies indicated that the passivated devices show a variation of the memristor resistive mechanism from SCLC to Schottky emission due to the reduction of interfacial defects.

The construction of 2D/3D MHPs heterojunctions on the surfaces of 3D MHPs represents a unique surface passivation process for the improvement of perovskite memristor performance. 2D perovskite could be introduced for recrystallization utilizing the defects of 3D perovskite films such as surface elemental vacancies and uncoordinated lead.^[^ ^174^ ^]^ The 2D layer contains hydrophobic organic cations, which can protect the 3D perovskite from external water and oxygen erosion. Meanwhile, the differences of the energy levels between 2D and 3D perovskites may effectively adjust the carrier transport capacity of the device.^[^ ^175^ ^]^ A 2D/3D MHPs heterostructure was formed by depositing n‐butylammonium iodide on the surface of MAPbI_3‐x_Cl_x_ by Xia et al.^[^ ^141^ ^]^ The 2D/3D MHPs heterostructure‐based memristor exhibited a significantly increase in the on/off ratio (≥10^3^) compared to the typical 3D MHPs devices, as shown in Figure 3b. The 2D/3D perovskite heterostructure facilitates the fabrication of grain homogeneity and highly dense structures. Furthermore, it passivates the defective states of MAPbI_3‐x_Cl_x_ films, thus improving the storage performance. Both Pan et al.^[^ ^160^ ^]^ and Lee et al.^[^ ^149^ ^]^ have modified the MHP surface by introducing PEA^+^ to fabricate devices with low SET voltage and high stability. It is noteworthy that both studies demonstrated that the MHP memristors, after 2D surface passivation, maintained their performance for a long period at room temperature and a certain humidity, as shown in Figure 3c for example. Additionally, Lee et al. also indicated that the prepared 2D perovskite film exhibits enhanced thermal conductivity compared to 3D perovskite, which could contribute to controlling the breakage of the CFs of the memristor.^[^ ^149^ ^]^

### 3.2 Crystal Modulation

Numerous researches have shown that the instability of perovskite materials is attributable to the presence of excessive GBs. These GBs are particularly susceptible to moisture due to the amorphous intergranular layer, which facilitates the rapid diffusion of hydrogen molecules into polycrystalline perovskite thin films along the GBs.^[^ ^176^ ^]^ Single crystal perovskite exhibits a markedly reduced number of GBs and charge trap densities relative to polycrystals,^[^ ^177^ ^]^ leading to better thermal and moisture stability of single crystal perovskite.^[^ ^178^ ^]^ Perovskite single crystals display a notable reduction in carrier concentrations due to low defects, which result in long depletion region widths and high Schottky barriers in metal‐semiconductor junctions. This phenomenon may have a positive effect on single‐crystal perovskite in memristor applications.

Li et al. indicated that CsPbBr_3_ single‐crystal perovskite memristors exhibit a Schottky hot‐ion emission mechanism at HRSs, demonstrating an exceptionally high switching ratio.^[^ ^99^ ^]^ They prepared Ag/CsPbBr_3_/Ag devices displaying a high on/off ratio of up to 10^9^, an endurance of 150, and a resistive state retention time exceeding 10^4^ s, as shown in Figure 3d. It was demonstrated that the higher Schottky barrier contributes to the reduction of the metal electrode and perovskite layer tunneling current, which is beneficial for subsequent large‐scale applications. Fernandez‐Guillen et al. have similarly indicated that single‐crystal‐based MAPbBr_3_ memristors outperform the polycrystalline counterparts.^[^ ^164^ ^]^ Moreover, besides the optimization in performance and stability, single‐crystal perovskite‐based memristors also display potential for the preparation of neural devices. Tian et al. implemented an ultra‐low‐power synaptic device using a 2D (PEA)_2_PbBr_4_ single‐crystal perovskite memristor, as shown in Figure 3e(I).^[^ ^105^ ^]^ They reported that the device has an ultra‐low HRS current of 10^−14^ and a switching ratio of 10 at a cycle count of 100, as shown in Figure 3e(II). Additionally, the devices could be operated at very low programmed currents, allowing for the achievement of multilevel storage with different current‐limiting settings (Figure 3e(III)). Finally, the simulation of synaptic behavior is completed, achieving ∼400 fJ/spike ultra‐low energy synaptic operation (Figure 3e(IV)), which is comparable to the energy expenditure of the human brain. Liu et al. subsequently prepared optoelectronic synaptic devices based on CsPbBr_3_ single crystals due to the excellent optical properties of the single‐crystal films, which expanded the synapse devices of perovskite.^[^ ^179^ ^]^ Similarly, Hou et al. utilized MAPbBr_3_ single‐crystals to prepare perovskite synaptic devices with high energy efficiency, operational stability, and temperature resistance in 2024. This report once again demonstrating that single‐crystal structures of perovskite are of great value for applications.^[^ ^180^ ^]^

### 3.3 Dimensional Optimization

The low‐dimensionalization of perovskites has been consistently identified as a potential solution to perovskite stability. The [BX_6_]^4−^ octahedral structure of perovskite is induced to crystallize in layered, long‐chain, clusters by the addition of different larger organic hydrophobic cations to form low‐dimensional perovskites. Compared to polycrystalline perovskite, low‐dimensional structures of perovskite exhibit a reduction in defects and GBs, and are also demonstrating the capacity to retain the intrinsic properties of 3D perovskite with additional quantum confinement effects.^[^ ^84^, ^181^ ^]^

#### 3.3.1 2D

The formation of layered structures is facilitated by the introduction of large organic ions, which serve to separate n layers of [BX_6_]_4_^−^ octahedra. Thus, the band gap and quantum confinement effects of the material can be modified by adjusting the value of n, which affects the optoelectronic properties, such as photoluminescence energy and carrier transport, and the band gap gradually increases with decreasing number of layers. Additionally, the incorporation of hydrophobic organic cations into MHPs can effectively improve the environmental reliability of the device, which has also been widely reported.^[^ ^182^ ^]^ By contrast to small ionic radius cations in MHPs with 3D structure, such as MA^+^, FA^+^, and Cs^+^, the larger size organic cations in 2D MHPs provide a barrier to the erosion of perovskite surface water.^[^ ^183^, ^184^ ^]^ Incorporation of a large hydrophobic cation into the 2D perovskite crystal lattice has been demonstrated to effectively suppress moisture corrosion to the perovskite interior.^[^ ^185^ ^]^ Extensive studies have been conducted on 2D perovskite‐based memristors, which have been shown to exhibit higher switching ratios and low resistance currents. Meanwhile, the 2D structure is beneficial to reduce the random perovskite defect movement during the growth and rupture of the CFs.^[^ ^147^, ^186^ ^]^

**Figure 4:**
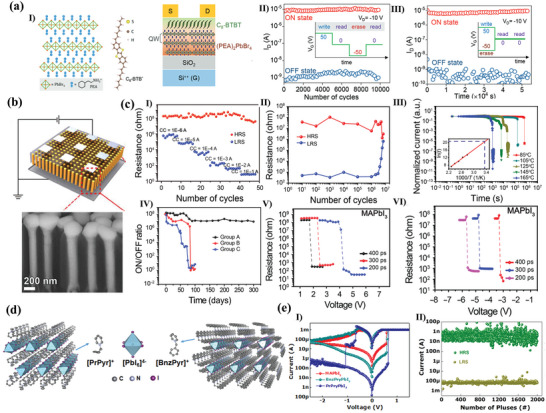
a, I) Illustration of the 2D layered perovskite PEA_2_PbBr_4_ and organic small‐molecule C_8_‐BTBT and schematic of a transistor incorporating the phase‐separated Blend‐II as the channel layer; II) Endurance performance of the PEA_2_PbBr_4_‐based memristor subjected to 10 000 continuous ON‐OFF programming cycles and (III) memory retention characteristics monitored for 55 000 s.^[^ ^144^ ^]^ Copyright 2021, John Wiley and Sons. b) Device schematic of MAPbCl_3_ NWs in the PAM template with Ag as active electrode and Al as counter electrode and cross‐sectional SEM image of MAPbCl_3_ NWs in PAM. c, I) Multilevel resistance states of Ag/MAPbCl_3_ NWs/ Al Re‐RAM devices with different current compliances; II) switching endurance performance of Ag/MAPbCl_3_ NWs/Al Re‐RAM devices; III) ON state retention time measurement of Ag/MAPbCl_3_ NWs/Al Re‐RAM devices at different temperature. Inset: ln(*t*)‐1000/*T* plot. Retention time at room temperature (RT) was extrapolated from the linear fitting; IV) reliability test of the Ag/MAPbCl_3_/Al Re‐RAM devices in an ambient environment and 85 °C/80% RH condition. Group A: Ag/MAPbCl_3_ NW/Al Re‐RAM device at ambient environment. Group B: Ag/MAPbCl_3_ NW/Al Re‐RAM device at 85 °C and 80% RH. Group C: Ag/MAPbCl_3_ thin‐film/Al Re‐RAM device at 85 °C and 80% RH; V) Writing speed and (VI) erasing speed measurements for MAPbI_3_‐based device.^[^ ^150^ ^]^ Copyright 2021, American Association for the Advancement of Science. d) Single crystal X‐ray structures of 1D lead‐iodide hybrids (PrPyr)[PbI_3_] and (BnzPyr)[PbI_3_]. e, I) *I–V* characteristics demonstrating the resistive switching effect in three different perovskites, MAPbI_3_, (PrPyr)[PbI_3_] and (BnzPyr)[PbI_3_]; (II) test results of AC endurance.^[^ ^166^ ^]^ Copyright 2024, Royal Society of Chemistry.

The group of Gedda reported a strategy to control the treatment of lamellar structures by Ruddlesden‐Popper phase perovskite film solutions based on PEA_2_PbBr_4_.^[^ ^144^ ^]^ The introduction of the organic semiconductors 2,7‐dioctyl[1]benzothieno[3,2‐b]benzothiophene (C_8_‐BTBT) into the perovskite formulation, was observed to promote the formation of large and nearly single‐crystal lamellar PEA_2_PbBr_4_ structures covered by ≈5 nm C_8_‐BTBT thin layer. The device comprising a PEA_2_PbBr_4_/C_8_‐BTBT channel exhibits an unexpectedly large hysteresis window between forward and reverse bias scan voltages. The combination of experimental results and theoretical calculations demonstrated that the phase enhanced by C_8_‐BTBT served as a hole‐transport channel. Meanwhile, the quantum well in PEA_2_PbBr_4_ could serve as a charge‐storage element, and carriers in the channel were injected, stored, or extracted through the tunneling. **Figure** **4**a(I) shows a schematic of the structure of the device. The modified devices exhibit high erase/write windows (≈10^4^), excellent data retention time (≈5.5 × 10^4^ s), and long endurance (>10^4^ cycles) (Figure 4a(II,III)). It is worth mentioning that this group has associated the transistor structure with the memristor, which provides a certain foundation for memristors in applications of integrated circuits and other applications. Subsequently, Kim et al. introduced large ionic radius cations (PEA^+^) into CsPbI_3_ to form a quasi‐two‐dimensional MHPs (PEA_2_Cs_3_Pb_4_I_13_) with an ON/OFF ratio (10^9^), which was three orders of magnitude higher than that of the CsPbI_3_‐based MHPs memristor.^[^ ^139^ ^]^ Due to the wide bandgap of PEA_2_Cs_3_Pb_4_I_13_, a high Schottky barrier was formed and the activation energy increased, which led to a decrease in HRS current. Similarly, there are many studies have demonstrated that the low resistive state currents of 2D MHPs memristor devices originate from the Schottky junctions formed at their interfaces.^[^ ^186^, ^187^ ^]^ In 2024, a study by Jang's team showed that perpendicular‐to‐substrate 2D MHPs memristors also have excellent performance. The flexible Ruddlesden‐Popper 2D perovskite memristor arrays prepared were able to maintain humidity stability for more than a year under bending conditions.^[^ ^188^ ^]^

#### 3.3.2 1D

The unique structure of 1D perovskite comprises rigid bulk organic cations that isolate the octahedral chains and carrier transport is confined to the chains, leading to higher exciton binding energies and prominent quantum confinement.^[^ ^189^, ^190^ ^]^ Since 1D perovskite has a good environmentally stable structure, previous studies in the field of perovskite solar cells have shown that the construction of 1D‐3D heterojunctions could reduce the interfacial defect density, improve cell efficiency, and enhance environmental stability.^[^ ^191^, ^192^ ^]^ The research based on 1D perovskite memristors has also gradually reported due to the advantages of light‐weight, portable, and wearable. In 2021, Fan et al.^[^ ^150^ ^]^ reported a memristor based on MHPs NWs array with the structure of Ag/MAPbCl_3_‐NWs/Al, as shown in Figure 4b. The large on/off ratio of ≈10^7^ obtained in MAPbCl_3_ NWs was utilized to obtain six distinct open‐state currents through adjusting compliant current, enabling multistage storage as shown in Figure 4c(I). The device also exhibited excellent endurance performance with a cycle life of up to 3 × 10^6^ cycles without any degradation in the on/off ratio (Figure 4c(II)). The unique NWs array structure of transversely passivated by porous alumina membrane (PAM), and the thermal reliability of MHPs memristor was improved, which resulted from the higher thermal conductivity of PAM than MHPs NWs. The current retention time of the device reached 7 × 10^5^ s when the device remained in the ON state at 85 °C, as shown in Figure 4c (III). Besides, the device could maintain an ON/OFF ratio of ≈10^7^ in normal environments (>300 days; Figure 4c(IV)). In extreme environments at 85 °C with humidity at 80%, the device could still maintain an ON/OFF ratio of ≈10^6^ for more than 85 days (≈2000 h). After a rigorous retention time study, the extrapolated data retention times were obtained from Arrhenius plots, the best estimated retention time was 28.3 years for the MAPbCl_3_‐based device. MAPbI_3_‐based device had the fastest write and erase response due to its lowest activation energy, as shown in Figure 4c(V,VI). The device was demonstrated to be capable of retaining data for long durations in high temperature and humid environments, which supported the long‐term environmental reliability of the MHPs memristor. In the same year, a MAPbI_3_‐based three‐dimensional array of quantum wires with dimensions close to the exciton Bohr radius was reported by Fan et al. again.^[^ ^75^ ^]^ They developed a device with a switching ratio of ∼10^7^ and a cycling endurance of up to 6 × 10^6^. Meanwhile, they stated that their devices exhibited the fastest switching speed (100 ps) currently which could be explained by involving fast‐moving electrons to reduce Ag^+^ cations in the body of the monocrystalline QWs. They also fabricated ultra‐compact storage units with a lateral size of ∼ 14 nm and a footprint of 153 nm^2^, with a 1‐bit effective storage area of 76.5 nm^2^, which was the smallest storage device at that time.

A research of 1D flexible propyl pyridinium lead iodide (PrPyr) PbI_3_ memristors was published by Mathews' group in 2021, which reveale the crystal structure is shown in Figure 4d.^[^ ^165^ ^]^ The device demonstrated the capacity to maintain a switching ratio of >10^5^ over 450 cycles with a retention time of ≈10^4^s. The main cryptographic device application they built in this work will be elaborated on later. And in 2024, they published a further study of (PrPyr)PbI_3_ again.^[^ ^166^ ^]^ They compared (PrPyr)PbI_3_ and benzyl pyridinium lead iodide (Bnz)PbI_3_ and found that due to the larger structure of PrPyr^+^ and Bnz^+^, they could only induce the formation of anionic polymerized PbI_3_^−^ strands, which may be related to coulombic interactions with positively charged pyridine rings. The absence of p‐p stacking in (PrPyr)PbI_3_ crystals was identified through characterization, and this p‐p stacking is an essential contributor to small‐molecule charge transport. The differences in mobility resulting from these structural variations contribute to the superior performance of the (PrPyr)PbI_3_‐based memristor showed a superior performance as shown in Figure 4d. The (PrPyr)PbI_3_ memristor exhibits a turn‐off current of ≈nA, an on/off ratio of ≈10^6^, a retention time of 10^5^s, and an endurance of 2000 (Figure 4e). They also constructed HP memory arrays with 50 000 devices across an area of 100 cm^2^ and 16 × 16 crossbars, the largest dot, point, and crossbar HP memory arrays to date. This provides further reference for an in‐depth study of the effects of dimensionality and compositional space on material properties and memristor performance, and also demonstrates that 1D perovskites have a remarkable potential for flexible integrated electronic devices.

#### 3.3.3 0D

**Figure 5:**
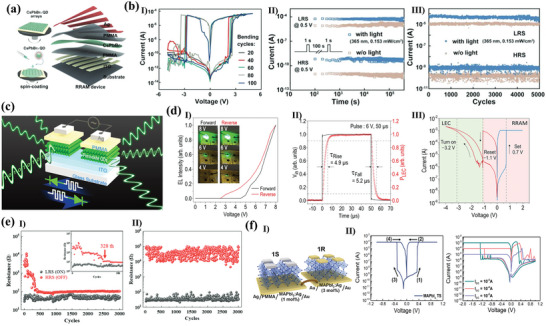
a) Schematic illustration of the CsPbBr_3_ QD‐based RRAM device fabrication via all‐solution process. b, I) *I–V* characteristics of the device at bending curvature of 15 mm radius with different bending cycles up to 100; II) Retention performance with and without light illumination. III) Endurance test results with and without light illumination.^[^ ^103^ ^]^ Copyright 2021, John Wiley and Sons. c) Schematic of the CsPbBr_3_ QD‐based LEM device composed of two nominally identical devices (top plot), with equivalent electronic circuit symbols illustrating that each device has dual functionalities either as RRAM or LEC, depending on the electric field direction across the LEM (bottom plot). d, I) EL intensity of the perovskite LEC versus sweeping voltage in a range of 0 V → 8V → 0V. Insets show the images of the LEC under forward (left) and reverse (right) voltages of 4, 6, and 8 V. II) Temporal P_LEC_ in response to a square‐wave pulse voltage of V_in_ (magnitude: 6 V, pulse width: 50 µs, and duty cycle: 50%) applied on the LEC. The rise (τ_Rise_) and fall (τ_Fall_) times of PLEC are also marked in the figure. III) *I–V* characteristic of the CsPbBr_3_ QD‐based RRAM under a dc bias sweep (0 V → +2V→ −4V→ 0 V). The set (0.7 V) and reset (−1.1 V) voltages of the RRAM mode, and the turn‐on (−3.2 V) voltage of the LEC mode are also marked in the figure.^[^ ^71^ ^]^ Copyright 2021, Meng‐Cheng Yen. e) Cycle endurance of the devices based on (I)ITO/MAPbI_3_/Al and (II)ITO/Y‐MAPbI_3_/Ag.^[^ ^143^ ^]^ Copyright 2021, Royal Society of Chemistry. f, I)The schematic representation of the MAPbI_3_:Ag‐based 1S‐1R structure. II) *I–V* behavior of the MAPbI_3_:Ag TS with bidirectional threshold switching characteristic following the sequence numbers and nonvolatile *I–V* behavior of the MAPbI_3_:Ag RS showing multilevel data storage capabilities.^[^ ^76^ ^]^ Copyright 2022, John Wiley and Sons.

As a 0D scale material, the quantum confinement effect of perovskite quantum dots provides a unique light absorption/emission efficiency.^[^ ^193^, ^194^ ^]^ The band gap can be modulated either through the control of the quantum dot size or the adjustment of the halogen element ratio, thus affecting the wavelength of its light emission.^[^ ^195^, ^196^ ^]^ Similarly, the size and halogen elements of perovskite quantum dots affect carrier transport. In 2018, Wang et al. reported CsPbBr_3_ QDs‐based flexible memristor arrays (**Figure** **5**a). The device and the *I*–*V* curves exhibited consistent behavior after 100 bending cycles, as shown in Figure 5b(I). They indicated that the devices they prepared had favorable photostability, and could maintain a switching ratio of ≈10^5^, an endurance of 5000 and a retention time of 4 × 10^5^ s an on/off current ratio under 365 nm, 0.153 mW cm^−2^ illumination (Figure 5b(II,III)). The authors indicated that the device exhibited superior performance and was capable of performing optoelectronic logic operations.^[^ ^103^ ^]^ Hao et al. similarly used CsPbBr_3_ QDs to prepare memristor devices, demonstrating synaptic behavior and modeling the visual system.^[^ ^197^ ^]^ An interesting study was reported by Yen et al. in 2021.^[^ ^71^ ^]^ They prepared a light‐emitting memory (LEM) device consisting of a light‐emitting electrochemical cell (LEC) and a memristor, which demonstrated a rapid response for the first time with CsPbBr_3_ QDs perovskite, as shown in Figure 5c. The external electric field was able to induce electric dipoles inside the perovskite and redistribute the internal ions toward accumulation at the interface to form a p‐i‐n diode, which facilitated photon emission through radiative complexation of electron‐hole pairs. The device exhibited enhanced reverse *I–V* characteristics and a stable optical hysteresis loop phenomenon with consistent wavelengths under reverse bias (Figure 5d(I)). The optical output power of a LEC modulated with a pulse bias was detected utilizing a photodiode. The results demonstrated that the LEC exhibited a light‐emitting response to pulses on a microsecond timescale (the extracted rise and fall times are ≈4.9 µs and ≈5.2 µs), as shown in Figure 5d(II). When a forward bias was applied, the device transition had a reliable electrical memristor with a retention of 10^5^ and an endurance of 10^4^ s. The transformation between the devices was dependent on both the bias polarity and the magnitude of the reverse bias voltage (Figure 5d(III)). This work demonstrates the unique properties of perovskite QDs and expands the application areas of research on perovskite memristors.

### 3.4 Doping Engineering

Doping has been extensively studied and applied as a principal methodology in the domain of semiconductor research. Perovskite, as an emerging semiconductor nowadays, has been similarly investigated to improve the device performance by introducing related impurities. Interestingly, the defect‐tolerant nature of MHPs prevents the addition of dopants from producing mid‐gap trap states.^[^ ^198^, ^199^ ^]^ This makes it possible to modulate the electronic and optical properties of MHPs devices by doping.^[^ ^200^ ^]^ In fact, the A, B, and X site elements in MHPs crystals are capable being doped, which consequently leads to numerous studies of perovskite materials in different fields, including different dimensions, organic‐inorganic and inorganic systems, and so on.^[^ ^201^, ^202^ ^]^ To simplify and prevent repetition, here we only discuss light doping and some special substitutional doping methods to optimize the perovskite memristor performance.

Luo et al. reported a memristor based on Y‐doped MAPbI_3_ as a resistive switching layer.^[^ ^143^ ^]^ The doped MAPbI_3_ film exhibited a smoother surface and more homogeneous particles compared to the undoped MAPbI_3_ film. It was indicated that doped Y could replace Pb atoms, thus changing the perovskite crystal orientation, modulating the energy levels and stabilizing the perovskite structure. Moreover, the modified device demonstrated not only maintained the non‐loss of storage capability, but also had a stabilizing resistive state of 3 × 10^3^ cycles, as shown in Figure 5e(I,II). The authors demonstrated that the doping effect could inhibit the quantity of defects and transform the conductive mechanism of HRS from SCLC to Schottky emission, which effectively improves the performance of the memristor. Jang's group prepared volatile threshold devices and nonvolatile memristor devices by adding Ag to MAPbI_3_ to form AgI doping.^[^ ^76^ ^]^ They demonstrated that the transition phenomenon between volatile threshold and nonvolatile memristor was determined by the concentration of doped Ag (Figure 5f(I)). At low Ag content, the devices exhibited characteristics consistent with diffuse memristors with volatile *I‐V* characteristics. Conversely, the devices displayed non‐volatile *I‐V* characteristics at high Ag content, as shown in Figure 5f(II). Characterization reveals that the crystallinity of the cubic MAPbI_3_ perovskite phase was enhanced and the grain size increased with increasing Ag concentration. The Ag‐doped MAPbI_3_ exhibits an increased grain size and more homogeneous orientation compared to the undoped MAPbI_3_. The doped volatile memristor had a switching ratio of 5.26 × 10^5^, a tolerance of 4 × 10^3^, and could respond within 80 ns. In contrast, the RS device demonstrated the ability to maintain a multistage resistive state for 1400 s. Finally, a selector‐a‐resistor (1S‐1R) integrated storage array was constructed utilizing the two devices. Similarly, Ma et al. reported the self‐assembly of spherical dendritic nanopolymers into 3D supramolecular framework (3D‐SF) by hydrogen bonding to modulate the flexibility, reliability, and RS behaviors of MHPs memristor.^[^ ^132^ ^]^ Therefore, the 3D‐SF network influenced the crystallization process of MHPs by ligand interactions with carbonyl, amide, and amine groups in perovskite precursors. With the assistance of 3D‐SF, the MHPs memristor could still operate for 500 cycles and maintain a switching ratio of 10^5^ under high‐temperature operating conditions of 90 °C. This study provides a convincing theoretical basis that the introduction of supramolecular framework in MHPs can guarantee the normal operation of memristor in challenging environmental conditions, such as high temperatures and humidity. Despite the existence of some MHPs materials without superior RS properties, it similarly provides a reference for subsequent research in the optimization of doping on MHPs memristors. Das et al. triggered the switching phenomenon by adding Cl^−^ to the perovskite structure due to the formation of iodine vacancies associated with the bridging of conductive channels.^[^ ^140^ ^]^ A series of RbPbI_3‐x_Cl_x_ films (*x* = 0, 0.3, 0.6, 0.9, and 1.2) were prepared. The obtained results indicated that the conductivity of the perovskite increased with the Cl^−^ concentration, accompanied by the emergence of structural defects, which resulted in an elevated amount of V_I_. Furthermore, c‐AFM measurements verified that undoped RbPbI_3_ film is intrinsically insulating, whereas Cl‐doped films exhibit resistive switching behavior.

### 3.5 Perovskite‐Like Compounds

There are also some compounds with perovskite‐like structures that have also been explored for the fabrication of memristors due to their structural similarity to perovskites. Although these compounds are not technically classified as ABX_3_ perovskite materials, most researchers refer to them as perovskites due to their resemblance in properties as perovskite materials. From this perspective, we will present a singular exemplar and observation, while continuing to utilize the term perovskite for ease of reference. Low‐dimensional lead‐free halide compound memristors based on Cs_3_Bi_2_Br_9_ were prepared by Bao et al.^[^ ^128^ ^]^ The crystal structure turned into isolated clusters of 2D perovskite due to the addition of Bi^3+^. The *I–V* characteristic curves of the devices showed adaptive characteristics and had a stable switching ratio of 10 after 3200 cycles, with a retention time of 10^3^ s. These findings suggest that the Cs_3_Bi_2_Br_9_‐based memristors had good environmental stability and remained memristive performance after 7 days of dark storage under ambient conditions (temperature 28 °C, humidity ≈40%). This provides a new reference for the realization of environmentally stable perovskite memristors. In addition to utilizing Bi‐substituted Pb, Park et al. also employed Sb‐substituted Pb to prepare MA_3_Sb_2_Br_9_‐based memristors.^[^ ^158^ ^]^ The MA_3_Sb_2_Br_9_‐based memristor has an endurance of 300 cycles, a retention time of ∼10^4^ s, and a multi‐level data storage capability. Also, the device is capable of low‐power (117.9 fJ µm^−2^) synaptic behavior. Although the performance of these perovskite‐based memristors is not very satisfactory at present, the non‐toxic nature of the substitutional Pb and the favorable environmental stability makes them a promising avenue for further research in the field of memristors.^[^ ^203^ ^]^

### 3.6 Conclusion

This section presents a summary of the effect of optimization strategies on the performance of memristors, with a focus on passivation of perovskite memristors, adjustment of the crystal structure, modification of the material dimensions and doping. Since the majority of perovskite memristor instability stems from the external water and oxygen erosion of the perovskite film through the GBs, enhancing device stability could be improved by affecting the crystallite size through passivation, reducing the material dimension to isolate water and oxygen with hydrophobic organic cations, or by preparing a single‐crystal perovskite. To achieve a large on/off ratio and better endurance, adjusting the energy level to obtain a suitable Schottky barrier could be considered. Furthermore, stable migration paths and low high‐resistance currents could also be obtained by dimensionally restricting the carrier transport direction and migration rate. Since each of these optimization approaches possesses distinctive characteristics and some are mutually reinforcing, it is difficult to accurately delineate the impact of these improvement strategies on memristor performance.

## 4 Intelligent‐Oriented Application Exploration of MHPs Memristors

Contemporary intelligent information devices are confronted with considerable obstacles pertaining to intricate network configuration, substantial data volume processing, and the secure transmission of data, in the context of the ongoing advancement of our society. As a combination of electronic devices with new structures and materials, MHPs memristors show tremendous potential in information processing. This section will provide an overview of the potential applications of MHPs memristors in the fields of logic operations, information encryption, artificial synapses, neural networks based on artificial synaptic properties, and bionic organs, to provide references for further applications.

### 4.1 Applications Based on Resistance State Variation

#### 4.1.1 Logic Operations with Multiple Types of Input Signals

**Figure 6:**
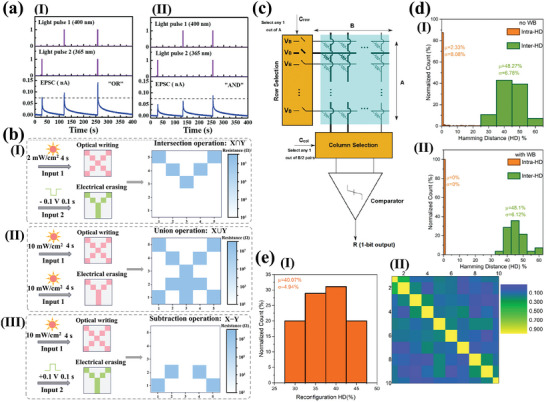
a, I)“AND” logic function achieved by two optical pulses (1 s, 0.3 mW cm^−2^) with the wavelength of 365 and 400 nm, respectively. and II) “OR” logic function achieved by two optical pulses (0.15 mW cm^−2^, 1 s) with the wavelength of 365 and 400 nm, respectively.^[^ ^205^ ^]^ Copyright 2021, John Wiley and Sons. b) Using photoelectric pulse to realize Boolean logic operation of image, in which the initial state of all the devices is LRS, the two images were input as “X” and “Y”. I) The mixed inputs of the weak intensity light stimulus and the negative pulse caused the resistive state of the superimposed region of the image changed LRS → HRS; II) Inputs are both bright light stimuli overlapping parts of the resistance state changed LRS → HRS; (III) The combination of strong light stimulation and positive pulses caused a change in the resistance state from LRS to HRS in the non‐overlapping regions of the two patterns.^[^ ^206^ ^]^ Copyright 2023, Molecular Diversity Preservation International. c) Circuit architecture of MHPs memristor PUF 32 × 32 crossbars. where consecutive device pairs in a row were treated as a differential unit, resulting in the generation of 512 independent device pairs, obtaining a randomness‐enhanced 512 bits. d) Inter/intra‐HDs histograms of the array (I) before and (II) after the write‐back strategy was adopted. The write‐back strategy reduces the BER from 2.33% to 0, while the UQ remains essentially constant. e) After 10 consecutive cycles of reconfiguration, I) HD histograms and correlation matrices were calculated for 45 pairs of bit streams. The average value of 40.07% indicated good independence for each cycle. II) The average value of cross‐correlation after reconfiguration was 0.2, indicating good independence between keys.^[^ ^165^ ^]^ Copyright 2021, Nature Publishing Group.

Distinct from conventional 0/1 logic switching devices, MHPs memristors have the property of modifiable storage space, making their realization of the logic operation storage function while expanding the state storage space.^[^ ^116^, ^128^ ^]^ As mentioned previously, perovskites have a space‐time dependence upon the input signal, making the utilization of pulse programming to implement a variety in logic operations possible. Meanwhile, as perovskites are an emerging photosensitive material, the researchers sensitively found that the introduction of illumination signals could induce the redistribution of halogen ion vacancies, promote the compounding of vacancies and ions, and enrich the logic operation of perovskite memristors. For instance, Sun et al.^[^ ^204^ ^]^ realized the “or” logic operation of optical/electrical signals on a simple memristor by introducing optical signals and coupling with electrical signals. Hao et al.^[^ ^205^ ^]^ adopted two different wavelength optical pulse signals as logic inputs directly, transferred the excitation modes through optical pulse power (0.3 and 0.15 mW cm^−2^), and finally implemented the “AND” and “OR” logic operations for the optical control (**Figure** **6**a). Furthermore, Zhang et al. expanded the logic gates implemented in a single device to a two‐dimensional plane, achieving the Boolean logic arithmetic function of the image.^[^ ^206^ ^]^ Since light stimulation promotes the redistribution of I^−^ and V_I_^+^, it makes the CFs break. The superimposed stimulation by light pulses with negative electrical pulses, optical pulses with optical pulses, and optical pulses with positive voltage pulses made the image matrix pixel points implement union, intersection, and difference set operations (Figure 6b).

#### 4.1.2 Physical level Encryption Device

As the scope of the Internet of Things (IoT) continues to expand in the context of everyday life, a considerable volume of user data is being transmitted by a multitude of mobile and edge devices through network infrastructure, making personal information security urgently demanded in the well‐developed network era. Although the software security systems employed in current encryption systems are indeed user‐friendly, the security systems are still at risk of being hacked and attacked by viruses, so physical‐level hardware encryption systems are considered superior comparatively. Among these, PUF represents a promising innovative primitive for authentication and key storage. In contrast to digital memory, PUF acquires information from the physical characteristics of the hardware, rather than storing secrets in digital memory. It follows that the question‐response pairs (CRPs) received by PUF and returned as outputs are unique. The performance of these systems can generally be evaluated based on four key metrics: uniformity (UF), uniqueness (UQ), reliability (REL), and reduced bit error rate (BER). UF is measured by the percentage of bits “1” or “0” in the response bit string, ideally 50%. UQ is determined by the inter‐Hamming distance (HD: in information theory, the Hamming distance between two equal‐length strings is the number of different characters in the corresponding positions of the two strings), which is defined as the HD between the responses of different PUF devices when subjected to the same challenge, where the ideal inter‐HD is also 50%. REL (100% ideal) is quantified using intra‐HD, which is defined by the HD between responses measured at different times and conditions on the same device for the same challenge. The BER is the normalized value of the intra‐frame HD, defined as the ratio of the number of erroneous bits to the response length.

In particular, the PUF based on memristors, which utilize the inherent randomness characteristics of hardware devices for encryption, has attracted the attention of scholars in the information security field and is expected to improve the security of hardware systems.^[^ ^20^, ^207^, ^208^ ^]^ The multiple switching physical properties of perovskite memristors (ion migration, self‐doping, electrochemical metallization, and local interfacial doping^[^ ^209^, ^210^ ^]^) allow for multidimensional entropy, which enables the generation of random and unique secret keys,^[^ ^211^ ^]^ making it impossible for intruders to clone PUFs without knowing the passwords. John et al. reported the first PUF secret key with 1Kb storage based on perovskites flexible memristor arrays.^[^ ^165^ ^]^ As illustrated in Figure 6c, they employed a differential circuit at the array output to eliminate the first‐order environmental dependence, reduce the temporal fluctuation of resistive/readout noise, and increase the sensing margins. In contrast to traditional techniques such as error correction codes with helper data to regenerate the secret keys within noisy channels and temporal majority voting, they adopted a write‐back strategy that saved a lot of time, area, and power costs as well as ensured PUF key to possessed more desirable UF (49.02%), UQ (48.3%), REL, and BER of the PUF. UQ/REL were measured by the inter/intra‐HD (Figure 6d), which represented HD between the responses of different/same PUF devices under the identical challenge, ideally UQ = 50% and REL = 100%. Moreover, under harsh operating conditions (time fluctuations, noise, temperature variations), the write‐back strategy could ensure that UF, UQ remain essentially unchanged, and BER≈0. The reconfigurable property of the PUF secret key contributed to the prevention of information leakage. They showed that using the HRS with a large coefficient of variation as the entropy source, and continuously configuring the key (conductance) was continuously configured by the periodical variation of the set‐reset sequence, which made the device irreversibly reconfigurable, that is, the CRP was completely different from the one before the reconfiguration, with good independence (Figure 6e). Conventional PUFs were vulnerable to deep learning algorithms that require only a handful of CRPs to predict all CRPs, even if the hardware could not replicate them itself.^[^ ^212^ ^]^ For more powerful PUF keys, they used a recursive algorithm to generate the keys, which could make deep learning computation on CRPs more challenging, and predicted that even with a 10 Mbps CRP collection rate, it would take 9.8 × 10^11^ years to reach nearly 100% attack accuracy.

### 4.2 Artificial Synapse

The process of learning and memory is an important part of the human ability to perceive the world, and it is also the most basic function of the brain. For a long time, researchers have been eager to develop a device with powerful computing power similar to the memory function of the brain, so as to further build a powerful analog circuit structure.^[^ ^90^, ^129^, ^213^ ^]^ However, based on traditional active/passive devices (e.g., CMOS), the complexity of the circuits makes them unfavorable for large‐scale applications.^[^ ^214^ ^]^ Intriguingly, the processes of ion immigration and resistance state modulation change inside memristors are strikingly similar to those of biological neurotransmitter conduction, which could make this component a key technology for enabling brain‐like computing.^[^ ^215^, ^216^ ^]^ Therefore, MHPs memristors with ion migration properties offer the possibility of mimicking the properties of biological synapses.^[^ ^217^, ^218^, ^219^ ^]^ MHPs memristors have been widely reported to simulate biological synaptic plasticity functions, such as paired‐pulse facilitation (PPF)/depression (PPD) and long‐term potentiation (LTP)/depression (LTD) functions, transformation of short‐term memory(STM) and long‐term memory(LTM), to assess memory duration and forgetting ability. At the same time, the complexity of neural activity was evaluated by simulating the functions of advanced plasticity of biological synapses, such as spike‐timing dependent plasticity (STDP), spiking‐rate‐dependent plasticity (SRDP, spike‐duration dependent plasticity (SDDP), spike‐voltage dependent plasticity (SVDP) and spike‐number dependent plasticity (SNDP).^[^ ^77^, ^220^, ^221^ ^]^ Briefly, short/long‐term memory is controlled by the quantity and spacing of pulses.^[^ ^222^, ^223^, ^224^ ^]^

**Figure 7:**
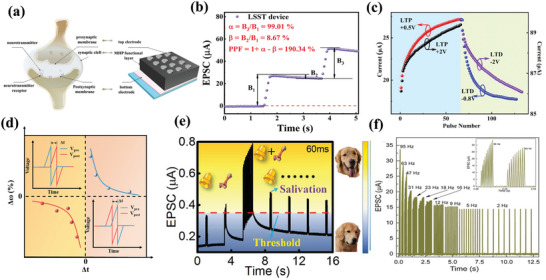
a) Schematic structures of biological synapses and MHPs artificial synaptic devices. b) PPF of the h‐BN‐optimized device under two continuous optical pulse stimulation (520 nm, 50 mW cm^−2^, 0.2 s).^[^ ^226^ ^]^ Copyright 2022, John Wiley and Sons. c) LTP/LTD plots of ITO/CsPbBr_3_/Au devices under 65 pairs with different amplitudes of positive/negative pulse stimulation, where the pulse amplitudes are +2 V, ‐2 V and +0.5 V, ‐0.8 V.^[^ ^159^ ^]^ Copyright 2023, Nano Materials Science. d) Schematic diagram of a typical STDP learning rule curve, where Δ*t* = *t*_pro_ − *t*_post_, when Δ*t* > 0, the presynaptic membrane is stimulated before the post‐synaptic membrane, thus a positive change in synaptic weights; when Δ*t* < 0, the presynaptic membrane is stimulated after the post‐synaptic membrane, thus a negative change in synaptic weights. e) The threshold current for the salivary secretory response was 0.35 µA, the pulse amplitude was 0.13 V, and the pulse width was 60 ms; the optical pulse power was 255 mW cm^−2^ and the duration was 60 ms.^[^ ^231^ ^]^ Copyright 2020, John Wiley and Sons. f) EPSC variation after applying 2–95 Hz pulse stimulation (each frequency sequence consists of ten +3 V pulses) to the device.^[^ ^234^ ^]^ Copyright 2018, John Wiley and Sons.

PPF and PPD are measures of instantaneous memory capacity and forgetting rate by applying two pulses (the same pulse height and width) to the device and continuously varying their pulse spacing (Δt). The PPF function is considered as the most significant simulated object in the simulation of biological synaptic function, due to its ability to respond to the process of memory reinforcement under different time intervals for stimulation. The properties of PPF are expressed by $PPF=100\%\times \frac{\left(A_{2}-A_{1}\right)}{A_{1}}$, in which A_1_ and A_2_ are the excitatory postsynaptic currents (EPSC) after the first and second pulse stimulation, respectively. While without stimulation, the current decays (analogous to the forgetting process) and consists of fast decaying phases and slow decaying phases. The decay process is expressed as $PPF\;=C_{0}\;+C_{1}\times \exp\left(-\Delta t/\tau_{1}\right)+C_{2}\times exp\left(-\Delta t/\tau_{2}\right)$, in which C_1_ and C_2_ are the initial promotion amplitudes of the fast and slow phases, respectively; the *τ*_1_ and *τ*_2_ are the characteristic relaxation times of the fast and slow phases, respectively.^[^ ^172^ ^]^ The research field of MHPs‐based memristors has been extended to biological neuromorphology, where the top and bottom electrodes of the MHPs memristor correspond to the presynaptic/postsynaptic membrane of the neuron, respectively, and the perovskite layer resembles the synaptic gap (**Figure** **7**a). Real‐time recognition/decoding of visual information is intimately related to PPF behavior of Postsynaptic Neurons.^[^ ^225^ ^]^ Excellent PPF performance is beneficial to the memristor to capture information quickly. After adding hexagonal boron nitride (h‐BN) to the CsPbBr_3_ quantum dot device, Wang's group used two continuous light pulses (520 nm, 50 mW cm^−2^, 0.2 s) to achieve PPF with an index of up to 196% (Figure 7b).^[^ ^226^ ^]^ The h‐BN hindered the complexation of photogenerated carriers between graphene and the CsPbBr_3_ quantum dot layer, which enables a higher EPSC subsequently based on the successful realization of real‐time sensing and storage.

LTP/LTD describes the excitatory/inhibitory response to successive identical positive/negative pulse stimuli, providing a scheme for the regulation of synaptic weight conductance. LTP/LTD, as a manifestation of long‐term memory/forgetting processes, is a necessary function for devices to become artificial synapses. As the quantity of pulses applied to the device increases, the more precise the weight modulation will be. Moreover, a wider conductance range allows for a larger adjustable ability and ultimately more information will be stored in the memristor.^[^ ^227^ ^]^ Meanwhile in several neural networks for image recognition (e.g., CNN), the recognition accuracy is enhanced as the linearity of the weight modulation improves.^[^ ^228^, ^229^ ^]^ Luo et al.^[^ ^159^ ^]^ discovered that LTP/LTD linearity could be altered by adjusting the continuous stimulus amplitude (two pulse amplitudes of ±2 V and +0.5 V, −0.8 V), after which synaptic devices with varying linearity were formed into weight matrices to identify neuronal outputs (Figure 7c). They indicated that the recognition rate of CNNs using low‐pulse stimulation of synapses could reach 96.7%. Zhang et al. revealed the effect of dimensional changes in MHPs memristor on synaptic plasticity.^[^ ^230^ ^]^ The results indicated that the PPF index of the 0D MHPs memristor was superior under the same stimulus. Meanwhile, relative to other devices (1D Cs_3_Bi_2_Cl_9_ and 2D Cs_3_Bi_2_Br_9_), the 0D Cs_3_Bi_2_I_9_ also exhibited lower conductance and more stable modulation process in LTP/LTD, which is conducive to lower power consumption and improved device stability.

The advanced synaptic plasticity enables more complex learning styles. In particular, the STDP is the basic and most common mechanism for learning memory, which simply means that postsynaptic currents exhibit excitatory/inhibitory behavior when a stimulus applied to a presynaptic neuron is earlier/later than a stimulus applied to a postsynaptic neuron, and that the temporal spacing of the two stimuli also tunes the strength of the postsynaptic currents (Figure 7d). The mathematical model of synaptic weight variation is simplified in computational neuroscience as Δω  =  A*e* ^−Δ*t*/τ^ + Δω_0_, where A and *τ* are the scaling factor and time constant of the STDP function, respectively, and ∆*ω*_0_ is a constant representing the nonunitary portion of the synaptic variation. The SRDP is ubiquitous in synaptic activity, reflecting the ability of varying stimulus frequencies to modulate the synaptic weights of neurons. Based on this, some complex learning processes can be simulated by the memristor, such as the most typical associative memory training case of Pavlov's dog, which simulates the rules of STDP and strengthens the connection between the two stimuli, even if the ringing of the bell can make the weights exceed the threshold of saliva secretion, and the subsequent loss of the results of the training with the “slack”. Figure 5e demonstrates the simulation of Pavlovian associative learning behaviors by a group of Jiang that applied ringing signals (electrical pulses) and food signals (light pulses) to a CsPbBr_3_‐QDs/MoS_2_ device.^[^ ^231^ ^]^ A single bell/food signal could/could not prompt a dog to secrete saliva, however a single bell signal also promoted salivation after the application of 10 combined training signals, enabling the simulation of biological associative learning by an artificial device. The SRDP, as an advanced extension to the PPF, is frequency‐dependent and can be used as a dynamic signal time‐domain filter to provide a basis for higher‐order spatio‐temporal identification functions.^[^ ^232^, ^233^ ^]^ Generally, the EPSC of the device increases with pulse frequency as shown in Figure 5f. Interestingly, the team of Mathews showed^[^ ^234^ ^]^ that different A‐site ionic MHPs memristors exhibited different relaxation times and filtering gains (ratio of EPSC amplitudes of the last and first pulses of each frequency, B10/B1) for different frequency pulse trains (consisting of ten +3 V pulses). Compared to the inorganic cesium‐based system (CsPbBr_3_ = 1.34, 95 Hz), the organic system at (MAPbBr_3_ = 2.92, FAPbBr_3_ = 1.74, 1.74 at 95 Hz) was more promising in the frequency domain. Furthermore, the MA‐based MHPs memristor prepared by Huang et al. demonstrated excellent filtering characteristics and realized the sharpening of flower images (cut‐off frequency of 19 Hz).^[^ ^235^ ^]^

### 4.3 MHPs Memristors Employed in Bionic Synapses and ANN

Early neuroscientists constructed a mathematical model called Artificial Neural Networks (ANN) to simulate brain functions. Nowadays, ANN has developed into one of the most important technologies in the intelligent information generation. The ANN simulates the connection of biological neurons through the interconnection of multiple nodes to form an adaptive nonlinear dynamic system. Since perovskite memristors can be used as artificial neural components, as mentioned earlier, numerous specialists have carried out a series of research on the construction of ANNs using MHPs memristors, including CNNs, Spiking Neural Networks (SNNs), etc.^[^ ^29^, ^236^, ^237^ ^]^

#### 4.3.1 Reservoir Computing with ANN

**Figure 8:**
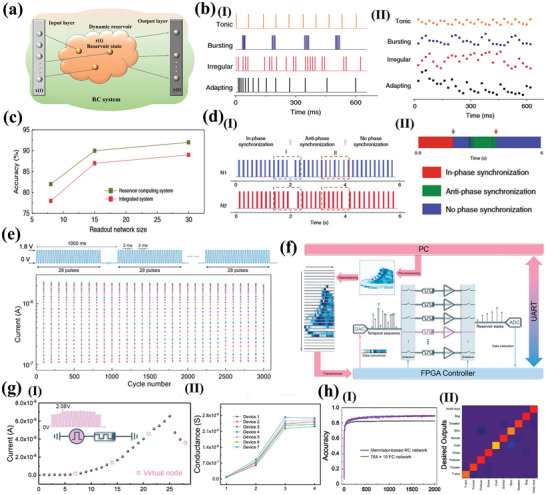
a) Schematic diagram of the RC system. The states of all neurons at moment t are mapped from the input function x(t) to the reservoir space of r(t) through nonlinear changes, then the different reservoir states are analyzed, trained, and finally the desired output y(t) is produced. b, I) Illustration of the spike firing pattern pulses of four neurons, and II) evolution of the memristor read current for the spike trains. c) Comparison of neural firing pattern recognition accuracies between the integrated system and the RC system with different‐sized readout layers at fixed time. d, I) Spike trains showing synchronization states evolving from in‐phase synchronization, anti‐phase synchronization to no‐phase synchronization. The gray bars mark the transition between different synchronization states. II) Classification results for the RC system. The arrows indicate the moment when a synchronization state transition is detected.^[^ ^78^ ^]^ Copyright 2020, Nature Publishing Group. e) Pulse schematic diagram and the endurance test of the memristor. Each 28 pulses is divided into a group, and a certain time interval is set between each group of pulses to allow the memristive conductance to spontaneously decay back to the initial HRS. Endurance test result of the device under the stimulation of 3000 cycles (contains 84 000 pulses). The experimental results were sampled at 100 group intervals. f) Schematic process flow of RC system for image classification. The computer transmits the data to the FPGA through a universal asynchronous receiver transmitter (UART). The FPGA controls the multiplexer to select a certain memristor. The FPGA then collected the generated dynamic reservoir state through the analog‐to‐digital converter module and transmits it back to the computer through the UART. g) (I) Current response of the memristor to an arbitrary impulse sequence. The data points marked with pink asterisks represent the virtual nodes to be sampled for training. II) Extracted virtual node data of seven different memristors in response to the same stimulation adopted in (I). h, I) Comparison of recognition accuracies between the memristor‐based RC system and the software‐based neural network after 2000 epochs of training on the fashion‐MNIST dataset. (II) Confusion matrix for experimental classification results of memristor‐based RC systems.^[^ ^80^ ^]^ Copyright 2022, American Chemical Society.

The concept of RC was originally proposed to tackle the problem of gradient vanishing or gradient explosion in Recurrent Neural Networks (RNNs) during back‐propagation training.^[^ ^238^, ^239^ ^]^ The RC is a dynamic system that projects nonlinearly processed input signals into a high‐dimensional space (reservoir state). The nonlinear transform maps the original features in the time domain to features of the reservoir state, followed by extraction of the features by a small trained linear neural network (readout layer) (**Figure** **8**a).^[^ ^240^ ^]^ In particular, the characteristics of RC with short‐term memory (the state is determined by the current and recent inputs) are highly analogous to the neural impulse signals with time‐domain coding properties, therefore, dynamic memristors can be applied to RC systems.^[^ ^241^ ^]^ Lu et al. succeeded introducing MHPs memristors as reservoirs into RC systems by utilizing the short‐term memory properties of CsPbI_3_‐based MHPs memristors.^[^ ^78^ ^]^ The introduction of the concept of virtual nodes expanded the scale of the reservoir pool, and furthermore, the operating state of the memristor at different times was sampled and recorded via defined time interval, which facilitated the study of neural activity in response to the stimuli. Four different pulse string stimuli (“Tonic”, “Bursting”, “Irregular”, and “Adapting ”) were set to detect the spike‐firing recognition pattern of neurons (Figure 8b). “Tonic” and “Bursting” denote low‐frequency and high‐frequency spike trains with the same interval, respectively, “Irregular” denotes instability of the stimulation signal, and “Adapting” corresponds to a peak sequence with increased interval. They showed that the two‐layer CNN (readout layer) with the RC system achieved an 87% overall recognition rate for all four neural firing models, and that the RC system has advantages over conventional integrated devices (Figure 8c). Meanwhile, they found that the system successfully identified neural synchronization states at different moments and detected transitions between different synchronization states (Figure 8d), demonstrating that the memristor‐based RC system could assist in investigating the correlation between different neuronal peaks.

Further, Han's group fabricated an RC system utilizing a MAPbI_3_‐based memristor, the device held a huge amount of storage space of more than 2 orders of magnitude over a modulation range of more than 3000 consecutive burst cycles (27 pulses in a string) (Figure 8e).^[^ ^80^ ^]^ They immediately constructed a system consisting computer, a Field‐Programmable‐Gate‐Array (FPGA) controller, memristor array, peripheral operational amplifiers, and control circuits to verify the RC system capabilities (Figure 8f). The Fashion‐Mixed National Institute of Standards and Technology (Fashion‐MNIST) dataset images were preprocessed with Python assembly language and sent to the FPGA. Then, the FPGA converted the gray value of the image into continuous voltages by analog‐to‐digital conversion. The 28 × 28 image was converted into 28‐dimensional vectors, and the image information was represented by a continuous pulse train. A simple RC system was accomplished by connecting virtual nodes with temporal characteristics (one virtual node was generated for each 7 pulses and was able to distinguish features between different inputs (Figure 8g) to output layer by random weight multiplication. Eventually compared with the neural network without RC, it was found that the RC system could effectively extract the data features and reduce the network complexity (Figure 8h).

#### 4.3.2 Image Recognition and Visual Neural Networks

**Figure 9:**
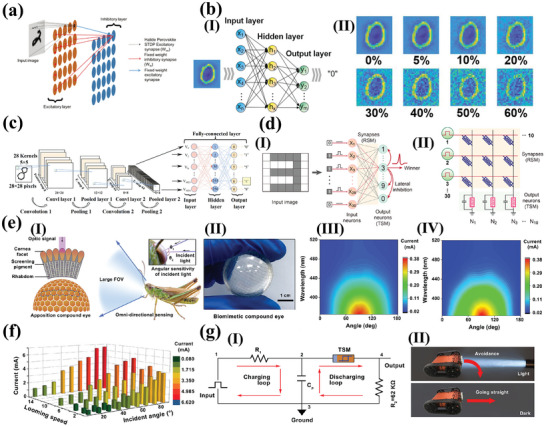
a) Schematic diagram of a bilayer neural network where inputs are connected to each excitatory neuron in first layer (layer 1) through synapses with STDP behaviors, and each inhibitory neuron in the second layer is connected to all layer 1 neurons through fixed‐weight inhibitory synapses, leading to a winner‐take‐all competitive relationship.^[^ ^234^ ^]^ Copyright 2018, John Wiley and Sons. b) Schematic diagram of a three‐layer ANN with a hidden layer, the output was connected to a differential circuit so that it still had a satisfactory recognition rate under a noise ratio of 0–60% (“0” as an example).^[^ ^243^ ^]^ Copyright 2023, American Chemical Society. c) CNN with two convolutional layers, two pooling layers, and one fully connected layer for handwritten digit recognition.^[^ ^159^ ^]^ Copyright 2023, Nano Materials Science. d) A two‐layer SNN model for 5 × 6 digit recognition, in which synapses and output neurons are composed of RSM and TSM, respectively.^[^ ^82^ ^]^ Copyright 2023, John Wiley and Sons. e, I) Detailed structure of the apposition compound eye of the locust with omnidirectional optical sensing function. The compound eye consists of thousands of integrated optical “ommatidiums” arranged spherically along a curved surface with wide field‐of‐view detection capability. II) Hemispherical bionic compound eye device and the conductance response to different wavelengths of incident light (III) in the *x* and (IV) *y* axis directions. f) Three‐dimensional spatial distribution of device current response to different incident light angles and different looming speeds. g, I) Schematic diagram of the circuit simulating the LGMD functional artificial neuron. II) Schematic decision‐making for the robot car with optic signal processing ability.^[^ ^250^ ^]^ Copyright 2021, Nature Publishing Group.

Nowadays, the ability to extract and identify target information efficiently and accurately is a critical measure for the reliability of an ANN. Since images could convey abundant information, image recognition is mostly chosen to verify the learning efficiency of neural networks. Generally, a neural network is divided into three parts: input layer, hidden layer and output layer. The input layer is responsible for importing data into the network, the hidden layer is able to transform the data into higher/lower dimensional values through the activation functions of the nodes, and the output layer outputs the desired values, each layer is connected by weights. Earlier neural networks based on perovskite memristors adopted simple single‐layer neural networks,^[^ ^51^ ^]^ double‐layer neural network^[^ ^234^ ^]^ (the double‐layer learning network shown in **Figure** **9**a as an example). Although the network scale was simple, the recognition rate on the MNIST data set of the network was almost around 80%, which could not be applied to complex situations. Tang et al. introduced a hidden layer into the fully connected neural network to introduce the data features into another dimension to achieve better linearization, and finally achieved a 94% recognition rate for MNIST.^[^ ^242^ ^]^ Additionally, the introduction of external circuits could also improve the recognition rate of the ANN. Huang et al. improved the recognition rate and anti‐noise immunity of the whole network by introducing a differential amplifier circuit into the output end of the three‐layer neural network with hidden layers (Figure 9b).^[^ ^243^ ^]^ With the advantages of local connectivity, weight sharing, and sampling dimensionality reduction,^[^ ^244^ ^]^ CNN could significantly reduce the redundancy and complexity in information extraction, and has a unique ability to handle images and other data with huge amount of information.^[^ ^245^ ^]^ Lou et al. constructed a CNN utilizing a CsPbBr_3_‐based memristor and achieved a 96.7% recognition rate for MNIST data (as shown in Figure 9c).^[^ ^159^ ^]^ The CNN consisted of two convolutional layers (24 × 24 and 8 × 8), two pooling layers (12 × 12 and 4 × 4), and one fully connected layer. The fully connected layer consists of an input layer (1024 neurons), a hidden layer (256 neurons), and an output layer (10 neurons). SNN, considered a third‐generation neural network, has similar capabilities to biological neurons, namely the ability to encode spatiotemporal signals as input signals. The asynchronous and parallel operations of SNNs make it possible to become a computational model for energy‐efficient neuromorphic hardware devices.^[^ ^246^, ^247^, ^248^ ^]^ The research group of Wang et al. demonstrated a two‐layer SNN network with resistive switching memristors (RSM) and threshold switching memristors (TSM).^[^ ^82^ ^]^ As shown in Figure 9d, the image “3” with a size of 5 × 6 pixels was input, and each pixel was sent to 30 RSM neuron nodes, which were controlled by a series of pulses. The output neurons based on TSM were fully connected to the input neurons through synapses based on RSM, and finally, a winner‐take‐all strategy was adopted to inhibit the transversal path (e.g., if the number is “3”, the neuron with output “3” is trained to discharge first, and the others are inhibited).

Benefiting from the unique optical properties of perovskites, perovskite memristors have the potential to be developed into visual neural network physical devices. Even more conveniently, light pulses can be set directly as input signals to the neural network. Yue et al. investigated the effect of pulse power, pulse duration, and pulse number on the recognition rate of ANN.^[^ ^249^ ^]^ They pointed out that with the increase in the number of pulses and laser power, the image recognition accuracy could be effectively improved. However, when the power density exceeded 1.06 W cm^−2^, the accuracy gradually reached saturation, and was forgotten in a longer time (15 s), and the recognition rate can still achieve more than 90%. Furthermore, the loss of accuracy was recovered after forgetting with only a small amount of training times, indicating that the device possesses excellent perceptual learning capabilities. Inspired by the effective collision prediction function of the lobula giant movement detector (LGMD) neurons (capable of firing peaks before collision), Su‐Ting Han et al. assembled a 20 × 20 TSM array and realized the bionic artificial compound eye of LGMD visual neurons utilizing CsPbBr_3_ QDs.^[^ ^250^ ^]^ They prepared a hemispherical memristor bionic compound eye mimic the locust compound eye function (Figure 9e(I)) and demonstrated that the device exhibited uniform light sensitivity in the *x* and *y* axes, with maximum response at incident light perpendicular to the device (shown in Figure 9e(II)), and was able to recognize wavelengths in the UV–vis region. The information captured by LGMD neurons for different looming object speeds (different incidence angles and different light intensities) as shown in Figure 9f. The results revealed that collision detection at 90° incidence angle was the most sensitive. The circuit shown in Figure 8g (I) simulated the function of LGMD neurons. The high and low resistances of the TSM were *R*_ON_ (LRS) ≈ 1 KΩ, *R*_OFF_ (HRS) ≈ 500 TΩ > *R*_1_, respectively, and C_P_ could be initially charged through the charging loop. Once the C_P_ voltage reaches the threshold, the TSM will switch from HRS to LRS, and C_P_ discharges through the discharge circuit, inducing artificial neuron to fire. LGMD neurons could convert optical pulse signals into peak signals (*f*_spike_) strongly dependent on the power of the light, the higher the optical power, the higher the *f*_spike_, and the spike signal amplitude gradually decreased after reaching the peak maximum. Finally, they employed the LGMD neurons to implement the collision avoidance function of an intelligent car. The neurons were resting and the car moved in a straight line without obstacles in front of it; when approaching an obstacle, the *f*_spike_ reached the threshold, and the neurons generated a spike signal that caused the car to turn (Figure 9g(II)).

#### 4.3.3 Nociceptive/Somatosensory Bionic Simulation

**Figure 10:**
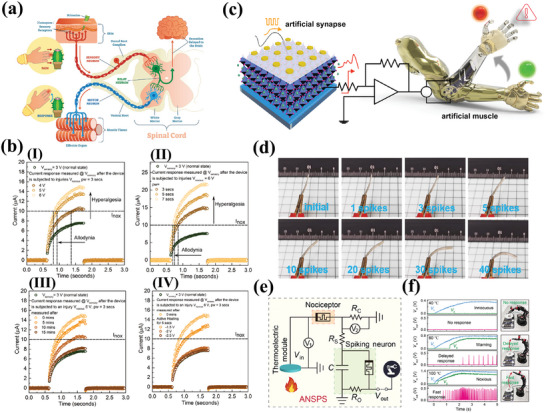
a) Schematic diagram of a biological pain receptor. An action potential is generated when the receptor receives strong stimulation, which transmits the information to the central nervous system for processing, causing the body to engage in escape behavior. b) Current response of nociceptors to different forms of stimulation. (I) Response to stimuli with different pulse amplitudes of the same pulse width (II) Increasing the pulse width of noxious stimuli to adjust the threshold of nociceptive sensitization (III) Applying noxious stimuli followed by noxious stimuli after varying relaxation times to investigate the duration of action of injury sensitization. (IV) Reverse voltage was applied after the application of noxious stimulus to simulate the healing process.^[^ ^254^ ^]^ Copyright 2021, John Wiley and Sons. c) A neuromuscular system based on perovskites artificial synapses and electrochemical artificial muscles. d) Electrochemical artificial muscle flexes progressively with an increasing number of spike stimuli.^[^ ^255^ ^]^ Copyright 2022, Nature Publishing Group. e) Schematic diagram of a power‐free artificial nociceptive signal processing system consisting of sensors, nociceptors, and spiking neurons, and a robotic arm. f) ANSPS responses to a harmless stimulus (40 °C), a warning stimulus (60 °C), and a noxious stimulus (100 °C), respectively. V_1_ and V_2_ represent the voltages measured at the labeled positions in (e).^[^ ^82^ ^]^ Copyright 2023, John Wiley and Sons.

The sense of touch is one of the important ways for biological organisms to perceive external information.^[^ ^251^, ^252^ ^]^ The successful simulation of biological haptic capabilities using artificial devices will expand the field of bionic and brain‐like computing applications, such as robots with sensory capabilities that can effectively plan their course of motion to avoid contact with obstacles.^[^ ^214^ ^]^ Nociceptors, as specialized sensory receptors, are able to alert the nerve center to avoid damage to the organism upon an injurious stimulus (as shown in **Figure** **10**a). Generally, nociceptive sensations are characterized by four features: “threshold”, “no‐adaptation”, “relaxation”, and “sensitization”. The presence of a “threshold” allows the receptor to determine whether the stimulus has reached a harmful threshold. “No‐adaptation” manifests as neurons remaining alert after a stimulus reaches or exceeds a threshold, emitting higher spikes if harmful stimulus occurs during the alert period, and entering a “relaxation” phase if no stimulus occurs for a long period of time until a normal state is returned, both functions allow the organism to gauge whether to avoid the source of the stimulus. The sensitization corresponds to the phenomenon of pain sensitization in the organism, where neurons reduce the pain thresholds after injury and a small stimulus produces intense pain to alert the organism to protect the area.^[^ ^202^, ^253^ ^]^ The Mathews team successfully prepared the diffusion memristor by adding poly(3,4‐ethylenedioxythiophene)‐poly(styrenesulfonate) (PEDOT:PSS) to the MAPbBr_3_ memristor to optimize the interface and simulate the relevant features of the injury receptor.^[^ ^254^ ^]^ They demonstrated that MHPs injury receptors respond to injury stimuli, including injury intensity tests (stimulus voltage amplitude), duration of stimulation (pulse width), nociceptive nonadaptive tests, and self‐healing ability (applying inverse voltage to inhibit neurons) (Figure 10b). Harmless stimuli under *V*_sensory_ ≤ 3 V, pulse width (pw) = 16 ms conditions did not cause neurons to emit warning and pain signals (*I*_warn_ = 8 µA, *I*_nox_ = 10 µA), nor did they trigger warnings when the stimulus was saturated. When *V*_warn_ = 4 V, pw ≥ 1.5 s, the elicited spiking signals caused by toxic‐like stimuli were higher than the warning/pain threshold output of the injury receptors. At the same time, the noxious damage pulses (*V*_sensory_ ≥ 4 V, pw ≥ 3 s) sensitized the neurons, and the sensitization current was measured under the harmless stimulus. The pressure sensing structure was integrated with the pain receptors and synapses into a peripheral signal processor, and the CMOS neuron circuit was integrated as a relay signal to accomplish the escape reflex function of the sensing robotic arm. This research, which utilized the ion diffusion properties of MHPs memristors to simulate notional stimuli, has inspired researchers to explore the potential for new applications of MHPs memristors. Xu et al.^[^ ^255^ ^]^ achieved pulse modulation of muscle deflection motion using an MHPs memristor (MAPbI_3_ passivated by PEAI) and an artificial muscle (Pt/Nafion membrane/Pt) (Figure 10c). Under the action of an electric field, the hydrated cations inside the Nafion membrane migrated to the cathode, causing the Nafion membrane to swell asymmetrically and deflect toward the anode. Modulation of the number of spikes (sensory information) stimulated artificial muscle activity, with muscle flexibility exceeding 90  at 40 pulses (Figure 10d). They also designed a neuromuscular fatigue warning system to warn overworked muscles. Wang et al. similarly tested four properties of nociceptive sensation utilizing CsPbBr_3_@graphdiyne‐based volatile TSM, and further constructed an artificial nociceptive signal processing system (ANSPS) without a power supply to investigate the perception of temperature changes (Figure 10e).^[^ ^82^ ^]^ A leaky integration–ignition circuit was used to convert continuous current signals collected from damaged receptors into pulse signals, and the thermal robotic arm was constructed to simulate the escape phenomenon of the organism stimulated under high temperature. A high output response was generated and delivered to the spike neuron at a voltage exceeding the threshold of the injury receptor (i.e., the harmful stimulus). The pulse signals generated by the pulse neurons were then sent to the motor controller to drive the robotic arm away from the heat source (Figure 10f) (**Table** **3**).

**Table 3:** Recent MHPs memristor‐based applications.

Device Structures	Synaptic plasticity	Application	Refs.
Si/W/Cs_3_Cu_2_Cl_5_/Ag–In–Zn–S/Ag	LTP, LTD, STM, LTM	Pavlov's dog, Image recognition‐91.36%	[256]
ITO/PMMA/ CsPbBr_3_/PMMA/Ag	/	Logical operation	[103]
ITO/Cs_3_Sb_2_I_9_/Al	SRDP	/	[155]
ITO/ Cs_2_AgSbBr_6_	PPF, STM, LTM	Logical operation	[155]
ITO/PEDOT:PSS/PrPyr[PbI_3_]/PMMA/Ag^a)^	/	Encryption device	[165]
Au/MAPbBr_3_‐SCTP/Au(lateral structure)^b)^	PPF, SVDP, SDDP, SNDP, SRDP	/	[257]
ITO/(PEA)_2_PbI_4_/MA_1−y_FA_y_PbI_3−x_Cl_x_/Au	STDP, SRDP	/	[258]
ITO/Cs_3_Bi_2_Br_9_ QD/Al	/	Logical operation	[116]
FTO/CH_3_NH_3_PbI_3_/Au	/	Image Boolean logic operation	[206]
ITO/Cs_2_AgBiBr_6_/PMMA/Ag	/	Logic operation	[259]
ITO/SnO_2_/α‐FAPbI_3_/[CNBmim]Cl/Au^c)^	PPF, LTP, LTD, STDP	Image recognition‐89.3%	[100]
ITO/Cs_3_Bi_2_X_9_(X = I, Br, and Cl)/Al	PPF, LTP, LTD, STDP	Pavlov's dog	[230]
ITO/Cs_3_Bi_2−x_Li_x_I_9−2x_/Ag	PPF, LTP, LTD, SVDP, SDDP, STDP	Image recognition‐94.25%	[243]
ITO/MAPbI_3_/Pd	/	mimic biolongical nociceptor	[260]
ITO/Cs_3_Bi_2_I_6_Cl_3_/Al	PPF, PPD, SDDP, STDP	Pavlov's dog	[261]
GNWs/CsPbBr_3_ QDs/Au(lateral structure)	PPF	Image recognition‐96.42%	[249]
ITO/CsPbBr_3_/Au	PPF, LTP, LTD, STD, STP	Image recognition‐96.7%	[159]
ITO/MAPbI_3_/Au	/	RC system Image recognition‐90.1%	[80]
ITO/PET/MAPbI_3_/PEAI/Au	PPF	Logic operation, Pavlov's dog, muscular‐fatigue warning system	[255]
ITO/PEDOT:PSS/pTPD/CsPbBr_3_ NCs/Ag^d)^	/	RC system Image recognition‐91.8%	[81]
Si/SiO_2_/MAPbBr_3_‐5%RhB/Pentacene/Au(lateral structure)^e)^	PPF, LTP, LTD	Morse codes	[262]
Graphene/h‐BN/CsPbBr+QDs (lateral structure)^f)^	PPF, LTP, LTD, LTM, STM	Image recognition‐91.5%	[226]
ITO/PEA_2_MA_n‐1_Pb_n_I_3n+1_/Au	PPF, PPD, SVDP, SDDP, STDP	Image recognition‐91.5%	[92]
FTO/MAPbI_3_/Ag	SRDP, PPF, PTP, STDP	/	[263]
Au/(PEA_)2_SnI_4_/Au	PPF, STM, LTM	/	[137]

^a)^ (propyl pyridinium lead iodide (PrPyr[PbI3]));

^b)^ (single‐crystalline thin platelets (SCTPs));

^c)^ (1‐cyanobutyl‐3‐methylimidazolium chloride ([CNBmim]Cl));

^d)^ (poly(N,N’‐bis‐4‐butylphenyl‐N,N'bisphenyl)benzidine (polyTPD));

^e)^ (Rhodamine B (RhB));

^f)^ (hexagonal boron nitride (h‐BN))

## 5 Prospects and Conclusions

In summary, for the demonstration of the RS mechanism of perovskite memristors, the most representative is the in‐situ PL technique, which visualizes the formation and breakage of conductive channels inside the device in a real‐time manner. Since perovskite materials have a wide range of applications, we analyze the advantages of perovskite‐based memristors and conclude that low‐dimensional perovskites currently exhibit superior memristor performance in response to recent reports. Subsequently, the applications of perovskite memristors are discussed, and due to their unique optical properties, the most promising and potential applications should be the applications in optical synapses and visual neural networks. Then, we provide an overview of the current challenges of perovskite memristors, including RS mechanisms, device stability, fabrication integration techniques, and neural synapses. Although in‐situ PL techniques are currently available, more in‐situ techniques will further advance the study of mechanisms. Electrochemical impedance tests and principal calculations will also lead to a deeper understanding of the RS mechanism. Long‐term stability is an important challenge for commercialization, and since perovskites are inherently environmentally sensitive, the development of perovskite memristors with excellent stability remains a focus of current research. At the same time, efforts to develop perovskite memristors with high integration, multifunctional devices compatible with traditional CMOS processes are also conducive to subsequent commercialization. In particular, the development of perovskite memristors with very low power consumption, excellent stability, and excellent light absorption is still needed in optical neuromorphic computing.

The applications of MHPs memristors in information storage and encryption technology, logic operation, biomimetic synapses, neural network computing, and other fields show remarkable potential in recent years. In this review, we summarize the RS mechanism, performance optimization strategies, and the status of multi‐disciplinary applications of MHPs memristors, as well as discuss their major research advances. With the development of characterization techniques, the understanding of the RS mechanism of memristors is gradually becoming clearer. Many reports have been published on the characterization of CFs of MHPs memristors with TEM, EDS, AFM, etc., and a recent one utilizing the in‐situ PL technique is even more remarkable owing to its ability to dynamically observe the formation and breakage of CFs. The low‐dimensional perovskite materials (1D and 0D perovskite) are presently demonstrating superior performance in the field of memristors, which show more competitive performance compared to other perovskite memristors, such as lower high‐resistance current, larger switching window, faster switching speed, and more stable performance. Furthermore, MHPs‐based memristors show significant potential at application prospects in information storage, data processing, flexible wearable devices, logic operations, biological synapses, and brain‐like neural morphology. Notably, the unique optical properties of perovskite materials make it easier to realize optical storage capabilities and optical synapse cells, which makes them highly promising for the realization of light‐based visual neural networks. However, the current application of MHPs memristors is still in the early stage of research, and there is still a long way to construct a complete system architecture. To that end, we will discuss the main challenges and our views on future research trends for MHPs memristors.

### 5.1 RS Mechanisms

So far, the RS mechanism of MHPs memristors reported mainly includes CFs model, interface RS mechanism, and charge capture/de‐capture model. However, these mechanisms have not been clearly distinguished, in particular, for the mechanism of multiple filaments competition/coexistence in carbon fiber models, there is no more direct characterization method to support this claim. In addition, the memristor may have multiple working mechanisms, which need further research and proof. The understanding of the mechanisms of MHPs memristors is conducive to the selection of memristor materials and the optimization of performance. At present, a variety of characterization methods have been used to study the mechanism of perovskite memristors. A work in 2024 showed the significant role of in situ PL technique in demonstrating the fracture of perovskite memristor filament formation.^[^ ^114^ ^]^ This suggests that in situ techniques can visualize the formation and destruction of conductive channels inside perovskite memristors. This study shows that the formation and destruction of conductive channels inside perovskite memristors can be visualized using in‐situ testing techniques. More in situ characterization techniques are expected to investigate the mechanism of resistance variation in perovskite memristors. Although there have been some reports of using electrochemical methods such as impedance mapping to study the ion motion inside MHPs memristors,^[^ ^129^, ^264^ ^]^ due to the complex ion‐electron system inside MHPs, a lot of work is still needed to gradually quantify and analyze the ion‐electron motion. Similarly, there are still few reports on first principal calculations of perovskite memristors, and more research on theoretical calculations of the mechanisms of perovskite memristors is still needed.

### 5.2 Long‐Term Reliability

For most commercial products, the retention time is expected to be at least 10 years, regardless of whether the device is operating or standby.^[^ ^265^ ^]^ Endurance provides an intuitive measure for the service life and working efficiency of memristors, even reaching 10^12^ for oxide‐based memristors.^[^ ^266^ ^]^ However, the MHPs memristor is still far from these standards. The long‐term reliability of the MHPs memristor still needs to be improved. Moreover, due to the instability of MHPs itself in high temperature and humidity environments, how to adopt effective strategies to enhance the environmental reliability of the device is an urgent issue to be solved. Due to the different crystal structures derived from different materials, as well as the different preparation methods, it is difficult to determine which material has the best memristor performance. However, for the time being, a lower‐dimensional approach does result in superior memristor performance. As the lower‐dimensional perovskite material has a quantum confinement effect, which limits carrier transport and results in a larger switching ratio, the device also has excellent environmental stability due to the presence of large organic hydrophobic cations. Although researchers have reported numerous strategies that could be beneficial to improve the environmental reliability of devices, these methods introduce cumbersome preparation processes with higher costs, which are not conducive to the commercial production of memristors. Thus, it is necessary to develop the MHPs memristor with novel structures and modification strategies. It is worth noting that the sensitive response of MHPs materials to illumination will be expected to achieve low‐power optoelectronic devices, which is a significant advantage to satisfy the practical demands, and requires continued efforts to be explored.

### 5.3 Challenges of Integration Device

MHPs memristor arrays have shown excellent performance in many applications. Nonetheless, the integrated density of these MHPs memristor arrays is low. Highly integrated devices with a small spatial volume and the ability to collect and process larger amounts of data need to be further studied. This requires researchers to continuously optimize the device fabrication process, explore and solve the problems of crosstalk between devices and structural damage by Joule heat in the case of high‐density integration. Meanwhile, there are only a few researches that simply integrate MHPs memristors with other types of devices (such as solar cells and detectors, etc.) to realize multifunctional applications, but most of the work is still based on the memristor itself and has not been integrated with other devices. Moreover, the fabrication of MHPs memristors differs greatly from the traditional CMOS process, which could be an obstacle to multifunctional integrated chips. With the deepening of the knowledge and research of memristors in science and industry, we boldly predict that the multifunctional integrated memristor devices will also become one of the research hotspots. Especially for the application of bionic synapses, it may lead to more abundant bionic devices. Integrating MHPs memristors with other types of electronic devices or directly constructing optical integrated devices‐based perovskites will greatly promote and expand the development of the memristor field.

### 5.4 Synaptic Devices and Visual Neural Networks

Although more applications of memristors are being explored, such as reservoir computing, encryption devices, etc., the most prominent feature of perovskite material is its unique light‐absorbing ability. The future trend of perovskite memristors is focused on optical devices. One of the most popular applications is to realize optical synapses and thus achieve a comprehensive simulation of biological vision. To simulate biological synapses, the first step is to reduce the power consumption of the device to the human brain level of ≈1–100 fJ per spike or even lower.^[^ ^267^ ^]^ At the same time, the synaptic device should have excellent consistency and stability for accurate signaling. Many groups are already investigating the possibility of developing biomimetic vision systems based on MHPs memristors.^[^ ^79^, ^250^ ^]^ The biological eye is composed of trillions of visual nerve cells, and some organisms are capable of capturing higher‐frequency flashes of information. In order to obtain a wider range of visual information and the ability to capture information about moving objects, it is necessary for vision devices to have a very high degree of integration, fast response to optical signals, and the ability to tolerate a large number of writes and erasures.

**Figure 11:**
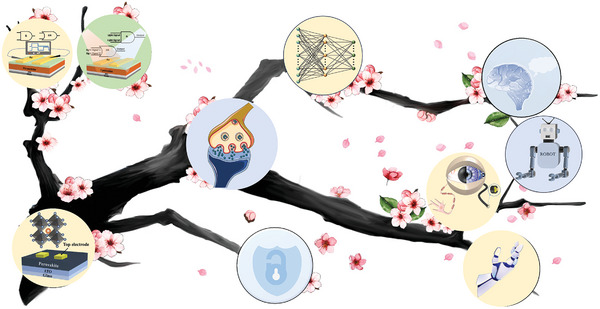
The development process and prospects of perovskites memristors.

We believe that with the endless efforts of researchers, the RS mechanism of MHPs memristors will be analyzed more thoroughly and its reliability will be improved. Furthermore, as more interdisciplinary researchers establish communications and collaboration, the MHPs memristors will also be more thoroughly researched for application in multiple fields. Perhaps in the foreseeable future (the development blueprint is illustrated in **Figure** **11**), brain computing, intelligent robots, electronic prosthetic, and so on in science fiction will appear in front of us.

## Conflict of Interest

The authors declare no conflict of interest.

## Author Contributions

B.J., X.C., and X.P. contributed equally to this work. The manuscript was written through the contributions of all authors. All authors have given approval to the final version of the manuscript.
